# Landscape of *BRAF* transcript variants in human cancer

**DOI:** 10.1002/1878-0261.70043

**Published:** 2025-05-25

**Authors:** Maurizio S. Podda, Danilo Tatoni, Gianluca Mattei, Alberto Magi, Romina D'Aurizio, Laura Poliseno

**Affiliations:** ^1^ Institute of Clinical Physiology (IFC), CNR Pisa Italy; ^2^ Oncogenomics Unit Core Research Laboratory (CRL), ISPRO Pisa Italy; ^3^ University of Siena Italy; ^4^ CTGLab, Institute of Informatics and Telematics (IIT), CNR Pisa Italy; ^5^ Department of Information Engineering University of Florence Italy

**Keywords:** *BRAF‐204*, *BRAF‐220*, cancer, KIRP, long reads, RNA sequencing, short reads, transcript variants

## Abstract

The BRAFV600E mutant kinase is widely studied as a cancer driver and therapeutic target. Here, we investigated how the annotation of the *BRAF‐reference* (*ref*) and *BRAF‐X1* variants has evolved in public databases and addressed challenges posed by their discrimination and quantification from short‐read sequencing. We built IsoWorm, a bioinformatic pipeline tailored to discriminate and quantify *BRAF* variants, and employed it to analyze > 600 cancer cell lines and > 1000 cancer tissue samples. Using FLIBase, we reanalyzed TCGA data from > 9000 cancer tissue samples. We consistently found that *BRAF‐X1* (now *BRAF‐204*) is very abundant in human cancer and its expression is 1.5–75 times greater than that of *BRAF‐ref* (now *BRAF‐220*). Crucially, we identified KIRP*‐kidney renal papillary cell carcinoma* as a cancer subtype in which a high *BRAF‐204/BRAF‐220* ratio is an independent prognostic factor of poor outcome. Our *in silico* analyses establish *BRAF* as a mix of two protein‐coding transcript variants, with *BRAF‐204* being more highly expressed than *BRAF‐220*. These findings prompt us to undertake the systematic benchmarking of *BRAF‐204* against *BRAF‐220* in terms of molecular mechanisms, biological activities, druggability, and clinical relevance.

## Graphical abstract

We investigate the annotation of *BRAF* variants, focusing on protein‐coding *BRAF‐220* (formerly *BRAF‐reference*) and *BRAF‐204* (*BRAF‐X1*). The IsoWorm pipeline allows us to quantify these variants in human cancer, starting from RNA‐sequencing data. *BRAF‐204* is more abundant than *BRAF‐220* and impacts patient survival. As ‘the real’ *BRAF*, *BRAF‐204* should be benchmarked against *BRAF‐220* regarding molecular mechanisms and clinical relevance

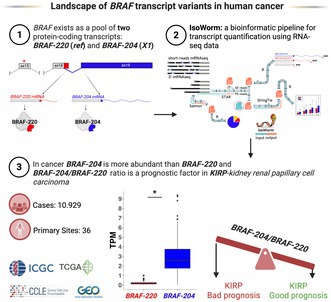

**Abbreviations**
ACC: adrenocortical carcinoma
ALL: acute lymphoblastic leukemia
BRAFi: BRAF inhibitors
BRCA: breast invasive carcinoma
C.N.S.: central nervous system
CCLE: cancer cell line encyclopedia
CDS: coding sequence
ceRNA: competing endogenous RNA
CESC: cervical squamous cell carcinoma and endocervical adenocarcinoma
CLL: chronic lymphocytic leukemia
CML: chronic myelogenous leukemia
COAD: colon adenocarcinoma
db: database
ESCA: esophageal carcinoma
FLIBase: full‐length isoforms in cancers and normal tissues database
FPKM: fragments per kilobase million
GBM: glioblastoma multiforme
GEO: gene expression omnibus
GTEx: genotype‐tissue expression
HCL: hairy cell leukemia
HR: hazard ratio
ICGC: International Cancer Genome Consortium
IQR: interquartile range
KICH: kidney chromophobe
KIRC: kidney renal clear cell carcinoma
KIRP: kidney renal papillary cell carcinoma
LAML: acute myeloid leukemia
LGG: brain lower grade glioma
LIHC: liver hepatocellular carcinoma
LUAD: lung adenocarcinoma
LUSC: lung squamous cell carcinoma
MANE: matched annotation from the NCBI and EMBL‐EBI
MESO: mesothelioma
NSCLC: non‐small cell lung cancer
OR: odds ratio
OS: overall survival
OV: ovarian serous cystadenocarcinoma
PCPG: pheochromocytoma and paraganglioma
PDPK1: 3‐phosphoinositide dependent protein kinase 1
PFI: progression‐free interval
POLR: proportional odds logistic regression
rel: release
SKCM: skin cutaneous melanoma
TCGA: The Cancer Genome Atlas
TGCT: testicular germ cell tumors
THCA: thyroid carcinoma
THYM: thymoma
TPM: transcript per million
TSL: transcript support level
UCEC: uterine corpus endometrial carcinoma
USC: uterine carcinosarcoma
UVM: uveal melanoma

## 1 Background

The RAF kinase family belongs to the ERK signaling pathway, which is involved in cell growth, differentiation, and survival. Approximately 60% of thyroid cancers, 50% of melanomas, 10% of colorectal cancers, and 3% of non‐small cell lung cancers exhibit a BRAF mutation at the V600 residue, which renders the kinase constitutively active. The BRAFV600E mutant kinase has been widely studied as a cancer driver and therapeutic target [1]. However, the regulation of *BRAF* gene expression and the full spectrum of its biological activities remain largely unexplored, although such knowledge is both valuable *per se* and instrumental for achieving more precise and effective targeting [2].

The expression of multiple isoforms from the same gene is extremely common in the human genome. It contributes to defining the complexity of higher vertebrates and maintaining their physiological state. However, it is also heavily altered in diseases, including cancer [3]. In our study published in 2017, we comprehensively investigated the landscape of BRAF mRNA and protein isoforms. We analyzed the 15 annotated and predicted RNA variants that were present in the Ensembl (release 80) and NCBI (GRCh38.p1) databases via RNA‐seq data from ~ 4800 cancer patients (9 cancer types) belonging to the TCGA. By mapping reads on variant‐specific exons or exon–exon junctions, we concluded that, in both healthy and cancerous tissues, *BRAF* mRNA invariably exists as a mix of three full‐length variants, namely, *BRAF‐reference* (*ref*) and *BRAF‐X1*, which we previously detected experimentally [4], as well as *BRAF‐X2*. These variants differ in the C‐term of their coding sequences, as well as in the sequence and length of their 3'UTRs. They also show expression level specificities, with *BRAF‐X1* being more highly expressed than *BRAF‐ref* and *BRAF‐X2* in most cancer cell lines, as well as in some of the cancer types analyzed. In terms of proteins, only BRAF‐ref and BRAF‐X1 are detectable, whereas BRAF‐X2 is unstable (its C‐term domain is recognized by the ubiquitin–proteasome pathway, and the protein is degraded) [5].

The ref and X1 isoforms have been further studied by our group, both as mRNAs and as proteins. We established that the *BRAF‐X1* variant is more phylogenetically conserved than the *BRAF‐ref* variant [5, 6]. Furthermore, we identified a group of microRNAs [7] and one mRNA binding protein (PARP1, [8]) that selectively bind the *X1* 3'UTR, positively or negatively affect *BRAF* stability/translation, and consequently contribute to fine‐tuning the output of the ERK signaling pathway. In terms of proteins, both the ref and X1 isoforms are endowed with kinase activity and together account for the known oncogenic features displayed by BRAFV600E in melanoma cells [5, 9]. Interestingly, melanoma‐free survival curves generated *in vivo* in adult zebrafish indicate that the BRAFV600E‐X1 protein is a much weaker melanoma driver than the BRAFV600E‐ref protein. Crucially, the presence of the 3'UTR quenches the oncogenicity of the ref protein, underscoring the importance of post‐transcriptional regulation and/or coding‐independent functions for BRAFV600E biology [6].

Since 2017, new versions of the NCBI and Ensembl databases have been released, up to the latest NCBI (GRCh38.p14) and Ensembl (113) databases; several RNA‐seq datasets of cancer cell lines (Cancer Cell Line Encyclopedia (CCLE, https://sites.broadinstitute.org/ccle/)) and tissue samples ((Gene Expression Omnibus (GEO, https://www.ncbi.nlm.nih.gov/gds), International Cancer Genome Consortium (ICGC, https://dcc.icgc.org/)) have become publicly available; the TCGA repository has been expanded to include data on approximately 11000 cancer patients (https://www.cancer.gov/ccg/research/genome‐sequencing/tcga/studied‐cancers); several computational methods for the identification and quantification of transcript variants from short‐read sequencing have been developed and benchmarked [10]; long‐read sequencing technology has proven its potential for variant identification, overcoming limitations intrinsically associated with short‐read technology [11, 12].

To provide an update on the identity and relative abundance of *BRAF* transcript variants in human cancer, here we describe the evolution of their annotation in publicly available repositories of RNA and protein sequences. We also quantify their expression levels across several datasets. We report that *BRAF‐X1* (currently *BRAF‐204*) is the predominant transcript variant in human cancer and it is expressed at a higher level than the *reference* (currently *BRAF‐220*) variant. We also highlight that the *BRAF‐204*/*BRAF‐220* ratio can be associated with a prognostic value.

## 2 Materials and methods

### 2.1 RNA‐seq data of cell lines and tissue samples

We analyzed:

- Raw paired‐end RNA‐seq FASTQ data of 690 cancer cell lines and 12 different tumor types (central nervous system (*n* = 64), colorectal (*n* = 56), hematopoietic and lymphoid (*n* = 176), kidney (*n* = 31), liver (*n* = 23), lung (*n* = 151), ovary (*n* = 42), pancreas (*n* = 41), prostate (*n* = 7), skin (*n* = 55), stomach (*n* = 34), and thyroid (*n* = 10)) from the CCLE collection;
- Raw paired‐end RNA‐seq FASTQ data of 762 cancer tissue samples and nine different tumor types (acute lymphoblastic leukemia (ALL, *n* = 173), acute myeloid leukemia (AML, *n* = 81), chronic lymphocytic leukemia (CLL, *n* = 29), chronic myelogenous leukemia (CML, *n* = 26), colorectal (*n* = 32), gliomas (*n* = 274), lung (*n* = 42), melanoma (*n* = 46), and prostate (*n* = 59)) belonging to nine GEO datasets;
- BAM files of aligned RNA‐seq reads of 258 cancer tissue samples and five different tumor types (esophagus (*n* = 7), kidney (*n* = 49), liver (*n* = 64), ovary (*n* = 60), and pancreas (*n* = 78)) belonging to five ICGC datasets. They were obtained as sliced BAM relative to the genomic region encompassing the *BRAF* gene (i.e., chr7:140718327–140926929, GRCh38.p14).

### 2.2 Data processing and *BRAF* quantification via IsoWorm

We built a bioinformatic pipeline called IsoWorm to process FASTQ or BAM files from short‐read sequencing experiments. IsoWorm is a Snakemake workflow that includes all necessary tools, supportive scripts, and config files for the automatic quantification of *BRAF* transcript variants. In more detail, raw paired‐end reads and metadata were downloaded from the SRA (CCLE and GEO samples) via the ‘ffq’ tool (v.0.2.1), which provided a list of SRA IDs. The FASTQ files were processed with salmon (v.1.10.2) and star aligner (v.2.7.1) using GRCh38.p14 as the FASTA reference genome/transcriptome and a GTF file with annotations of all annotated transcripts (Ensembl 113). Salmon generates TSV files with transcript quantification data according to the provided annotation, whereas the aligned BAM files produced by star were further processed with stringtie (v.2.2.1) via a custom GTF file that included only the genomic coordinates for the unique regions distinguishing *BRAF‐204* (i.e., chr7:140725145–140719327) and *BRAF‐220* (chr7:140732564–140730665) (Fig. S1). The sliced BAM files (ICGC samples) were downloaded with the ICGC score‐client API (v.5.8.1) and processed only with StringTie and the custom GTF file. IsoWorm is available at https://github.com/ctglab/isoworm.

### 2.3 Quantification of *BRAF* transcript variants

Through the IsoWorm workflow, we obtained two different variant estimations: one for all the 21 annotated variants via Salmon and the second specifically restricted to *BRAF‐204* and *BRAF‐220* via the GTF custom approach. In the latter approach, we calculated the FPKM for the two regions of interest, exploiting only the information (i.e., length and coverage) that is available from a sliced BAM, as we did for ICGC samples. We used the following formula:

$$\mathrm{FPK}M_{i}=\frac{q_{i}}{l_{i}*\sum_{j}q_{j}}*10^{9}$$

where $q_{i}$ is the raw fragments count specifically mapping unique genomic regions for *BRAF‐220* or *BRAF‐204*, $l_{i}$ is the respective genomic length, and $\sum_{j}q_{j}$ is the total number of mapped fragments (library size) retrieved from STAR log files. All the statistical tests were conducted via r (v.4.2.1).

### 2.4 Analysis of poly(a) sites

Raw single‐end QuantSeq 3′ mRNA‐Seq REV FASTQ data and the metadata of 97 lung cancer samples were aligned via IsoWorm as described above [13]. The obtained BAM files were processed through a custom R script via GenomicAlignments (v.1.34.1) and bedtoolsr (v.2.30) libraries to detect polyA sites at the 3'UTR. For the detection of the 3'UTR peak region in each sample, the following criteria were considered: (a) a coverage greater than 5% of the most highly expressed peak [14] and (b) a region length of at least 80 bp. All the peaks were compared to each other to select only those that were present and conserved in more than 50% of the samples.

### 2.5 Visualization of the expression and the ratio of transcript variants

All the plots of the expression profiles of the variants and their ratios were calculated and plotted via the r packages: data.table (v.1.14.2), dplyr (v.2.2.1), ggplot2 (v.3.3.6), tidyr (v.1.2.0), ggsignif (v.0.6.4), and patchwork (v.1.1.2). The TPM and FPKM values are represented in the plots as log2(TPM + 0.01) and log2(FPKM + 0.01), respectively.

### 2.6 Analysis of TCGA patient data

In addition to transcript expression levels, the clinical data of TCGA patients were obtained from the FLIBase web server (v.1) [15]. We selected the 11 cancer types that are characterized by the highest frequency of *BRAF* alterations [16]: ACC‐adrenocortical carcinoma, COAD‐colon adenocarcinoma, ESCA‐esophageal carcinoma, GBM‐glioblastoma multiforme, LUAD‐lung adenocarcinoma, KIRP‐kidney renal papillary cell carcinoma, OV‐ovarian serous cystadenocarcinoma, SKCM‐skin cutaneous melanoma, TGCT‐testicular germ cell tumors, THCA‐thyroid carcinoma, and UCEC‐uterine corpus endometrial carcinoma. For all tumors, patients were grouped according to their *BRAF‐204/BRAF‐220* ratio, creating three groups: Group 1, with the lowest ratios (≤ 33rd percentile (1Q)); Group 2, with mid‐range ratios (> 33rd percentile and ≤ 66th percentile (2Q)); and Group 3, with the highest ratios (> 66th percentile (3Q)).

We subsequently analyzed if Group 1/2/3 has an impact on survival time. We performed univariate Cox proportional hazards regression (Table S1), adding the Benjamini–Hochberg (BH) correction for multiple comparisons.

For KIRP, we also performed multivariate Cox proportional hazards regression analysis, considering as independent risk variables not only Group 1/2/3, but also tumor stage (TNM I, II, III–IV), and the individual parameters that compose it: size and extent of the primary tumor (T3–T4/T1–T2), involvement of regional lymph nodes (N1–N2/N0), and presence of distant metastases (M1/M0).

Univariate and multivariate Cox proportional hazards regression analyses were conducted in r (v.4.2.1) using the survminer 0.4.9 and survival (v.3.5) Bioconductor packages.

To evaluate whether having a higher ratio increases the probability of being at a higher tumoral stage, we performed a proportional odds logistic regression for ordered category outcome analysis, considering stage I KIRP patients as a reference and using tumor stage (I < II < III–IV) as a response variable. The latter analyses were conducted via the mass (v.7.3.60) package in r.

The Brant Wald test was used to evaluate the model, and the results are reported in Table S2.

### 2.7 Analysis of *BRAF‐204* ceRNA network in the KIRP dataset at TCGA

The correlation between the expression levels of *BRAF‐204*, the transcripts of other genes, and microRNAs was investigated using Spearman's rank coefficient on the entire KIRP cohort (*n* = 290). The quantification of the *BRAF‐204* transcript was retrieved from the FLIBase database. The quantification of the transcripts of other genes (including *ENSG00000140992.18*/*PDPK1*) was obtained directly from the TCGA dataset using the recount3 library in r. The quantification of miRNAs was obtained from the GDC TCGA project. Transcript expression levels were expressed as log2(TPM + 1), while miRNA expression levels were expressed as log2(RPM + 1).

The Spearman's correlation coefficient (rho, R) ranges from −1 to 1 and indicates the strength and direction of the correlation: positive values for direct correlation and negative values for anticorrelation. All *P*‐values were corrected using the Benjamini–Hochberg (BH) correction for multiple comparisons. Statistical significance was defined as adjusted *P*‐values (*P*adj) < 0.05.

## 3 Results and Discussion

### 3.1 BRAF‐ref/220 and BRAF‐X1/204 isoforms in publicly available repositories of RNA and protein sequences

We have monitored the evolution of the annotation of ref and X1 isoforms of BRAF since 2017.

In 2017, we referred to the annotations reported in NCBI (GRCh38.p1) and Ensembl (80) databases, and we defined *BRAF‐ref* as the transcript corresponding to ID NM_004333.4 in NCBI and ID *ENST00000288602/BRAF‐001* in Ensembl. The two annotations are concordant in terms of CDS length (766 aa) but discordant in terms of 3'UTR length (590 nt in NCBI vs 121 nt in Ensembl). Our analysis of TCGA RNA‐seq data suggested that in human cancer the *BRAF‐ref* 3'UTR is even shorter than that reported in Ensembl and rarely exceeds ~ 76 nt [5].

The *BRAF‐X1* transcript variant was named after the description associated with ID XM_005250045.1 present in NCBI (‘PREDICTED transcript variant X1’). It is characterized by a 767‐aa‐long CDS and a lengthy 3'UTR (7160 nt), which, according to the analysis of TCGA RNA‐seq data, is fully transcribed [5].

As mentioned in the Introduction, the 3'UTRs of *BRAF‐ref* and *BRAF‐X1* mRNAs differ not only in length but also in sequence, whereas the proteins they encode for differ in the C‐term (ref: ‐GYG**AFPVH** vs. X1: ‐GYG**EFAAFK**). This is because the *ref* variant is composed of 18 exons, whereas the *X1* is a splicing variant encompassing a shorter version of exon 18 that is joined with a very long downstream 19th exon [4, 5, 17]. Interestingly, in melanoma samples, the 19th exon is included in *BRAF* gene amplification [18], as well as in the duplicated kinase domain that confers acquired resistance to BRAF/MEK inhibitors [19].

We tracked IDs and associated sequence details for the *BRAF‐ref* and *X1* transcript variants from 2017 to the latest release of the NCBI (GRCh38.p14) and Ensembl (113) databases. Currently, NCBI lists 25 *BRAF* transcript variants, whereas Ensembl lists 21 *BRAF* transcript variants.

**Fig. 1:**
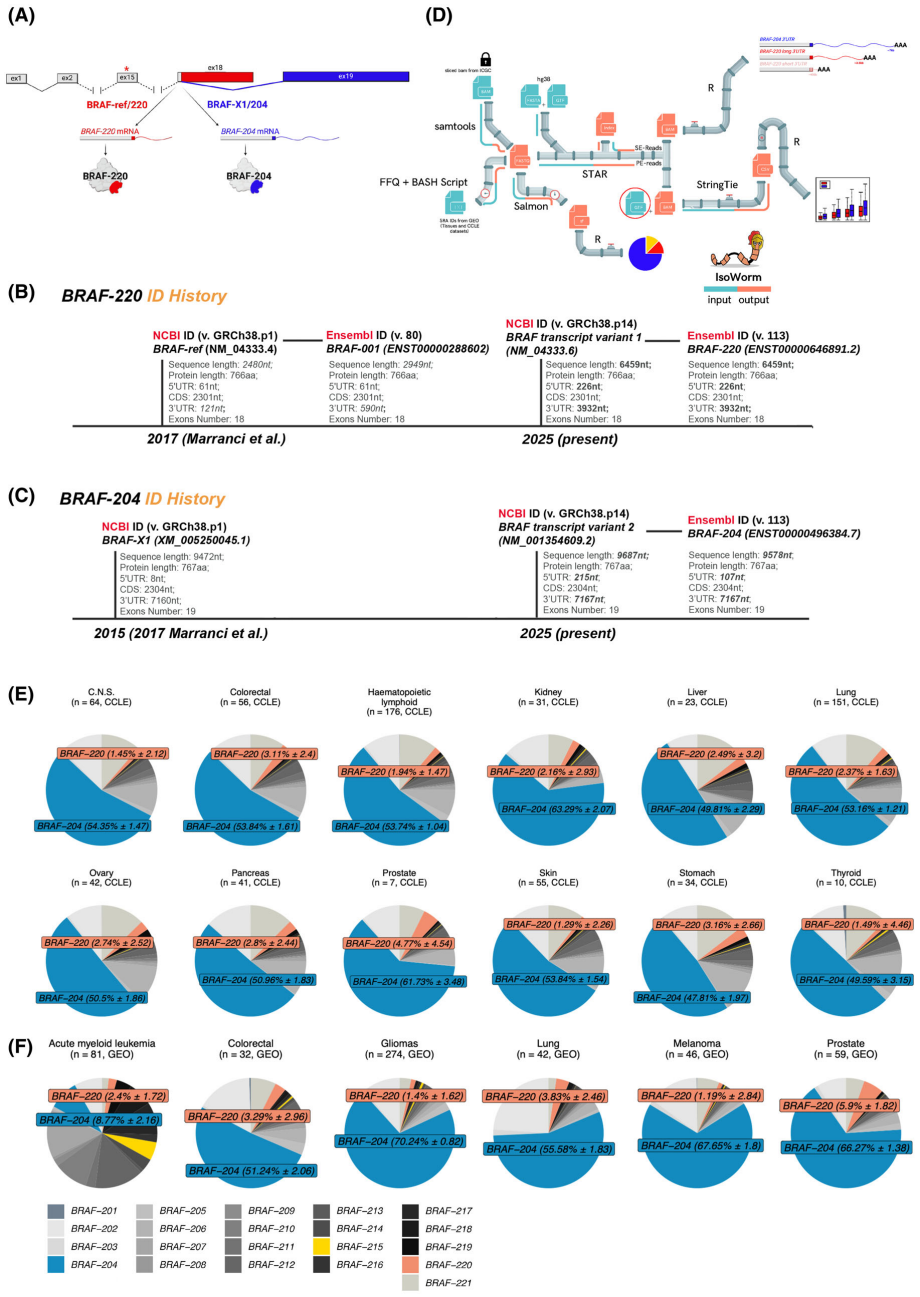
*BRAF‐204* transcript variant is highly expressed across cancer cell lines and tissue samples. (A) Cartoon summarizing the similarities and differences between *BRAF‐ref/220* (red) and *BRAF‐X1/204* (blue) RNA and protein isoforms. See the text for details. (B, C) Tracking of *BRAF‐220* annotation (B) and *BRAF‐204* annotation (C) from 2017 [5] to date. (D) Scheme of IsoWorm, the Snakemake pipeline that we developed to quantify *BRAF‐220* and *BRAF‐204* expression levels in large RNA‐seq datasets (both single‐ and paired‐end short reads). The pipeline consists of a series of interconnected modules that perform various stages of data analysis. It starts with a .txt file containing SRA IDs, while the indications about the RNA‐seq library type, or a BAM file, and the reference files (FASTA and GTF) are in the Snakemake config file. The custom module of IsoWorm was used to analyze the *BRAF‐204* and *BRAF‐220* variants specifically, and custom gtf files (red circles) were used to quantify variant‐specific genomic regions. The quantification was performed via Stringtie, and all the plots were generated with R language. Conversely, the Salmon module of IsoWorm was used to quantify the 21 transcript variants annotated in Ensembl db (113). An R script generates pie charts for *BRAF* variant expression. For single‐end reads, a module identifies polyA sites via custom R scripts. (E, F) Pie charts showing the 21 transcript variants of *BRAF* present in Ensembl (release 113) in both cell lines (E) and tissue samples (F). The mean relative abundance (%) of the 21 *BRAF* variants was estimated via the Salmon module of IsoWorm in 690 CCLE cell lines belonging to 12 cancer types and in 534 GEO tissue samples belonging to six cancer types. *BRAF‐204* is highlighted in blue, whereas *BRAF‐220* is highlighted in red. Mean ± SD values are indicated.

*BRAF‐ref* corresponds to ID NM_004333.6 present in NCBI and ID *ENST00000646891.2/BRAF‐220* present in Ensembl. The two sequences are identical and converge into a high‐quality annotation variant. In the MANE (Matched Annotation from the NCBI and EMBL‐EBI) database (v.1.3), the *BRAF‐220* ID is recognized as the ‘MANE Select’ transcript for the *BRAF* gene [20]. Compared with 2017, NM_004333.6 and *ENST00000646891.2/BRAF‐220* present longer 5′ and 3′ untranslated regions, such that the nominal length of the transcript exceeds 6 kb (Fig. 1A,B).

*BRAF‐X1* corresponds to ‘BRAF transcript variant 2’ present in NCBI with ID NM_001354609.2, while the matching Ensembl ID is ENST00000496384.7/BRAF‐204. Compared with 2017, these two annotations reveal slightly longer 5′ and 3′UTRs (Fig. 1A,C).

Notably, *BRAF‐X2* (XM_005250046.1 in NCBI (GRCh38.p1)) is no longer present (record removed).

In compliance with the most updated official nomenclature, from this point onward, we refer to *BRAF‐ref* as *BRAF‐220* and to *BRAF‐X1* as *BRAF‐204*.

A consistent annotation is not the only issue related to mRNA variants because for many transcripts the evidence of translation is very limited. The APPRIS database [21], which was developed within the GENCODE consortium and is used by GENCODE curators to update gene models, establishes principal variants via protein structural information, functional features, and cross‐species conservation. The current release of the db (2024_03v.49 Gencode47/Ensembl113) contains 11 variants for *BRAF*. None of them are selected by the APPRIS core modules as clear principal variants, and none are associated with strong proteomics. Nine variants are classified as *MINORs*, with a very low chance of being translated into (functional) proteins. The remaining two are *BRAF‐220*, which is classified as *PRINCIPAL:4* and has the highest TRIFID functional score (= 1), and *BRAF‐204*, which is classified as *ALTERNATIVE:1*. This is because it is flagged as TSL = 5 (i.e., without a single transcript that supports the model structure). However, *BRAF‐204* has a remarkably high TRIFID score (0.993), which in turn indicates that its likelihood of being functionally relevant and under selective pressure is very high.

The recently published FLIBase (Full‐Length Isoforms in cancers and normal tissues database, http://www.flibase.org), which annotates full‐length splice variants by combining long reads for discovery and short reads on exon–exon splice junctions for validation [15], identifies both *BRAF‐220* and *BRAF‐204* as full‐length and full‐splice variants (*TCONS_00952581* and *TCONS_00952571*, respectively). It is conceivable that *TCONS_00952571* will provide the experimental support necessary to increase the TSL of *BRAF‐204* in future releases of the NCBI, Ensembl, MANE, and APPRIS databases.

Finally, proteomic evidence, curated by CNIO and collected in the APPRIS database [21], confirms what we reported in 2017 [5]: isoform‐specific C‐terminal peptides were retrieved for both CCDS87555.1/BRAF‐220 (753–766aa‐TPIQAGGYGAFPVH (3 times)) and CCDS5863.1/BRAF‐204 (753–767aa‐TPIQAGGYGEFAAFK (15 times)).

In conclusion, *BRAF* should be fully acknowledged as a mix of *BRAF‐220* and *BRAF‐204* transcript variants, which differ in length and sequence of their 3'UTRs and are translated into protein isoforms that differ at the C‐term.

### 3.2 Tools for the quantification of *BRAF* transcript variants

Even though the official annotation for *BRAF* transcript variants has been updated (see above), the precomputed quantification reported in various databases (XENA, https://xenabrowser.net/; GEPIA2, http://gepia2.cancer‐pku.cn/#index; SRTdb, http://www.shenglilabs.com/SRTdb/) is still based on an older Ensembl release (version 40). Specifically, *BRAF‐220* is present as the old *ENST00000288602/BRAF‐001* transcript, whereas *BRAF‐204* is missing.

To quantify the *BRAF‐220* and *BRAF‐204* transcript variants in the TCGA datasets, we used the above‐mentioned FLIBase [15]. By combining datasets produced by both short‐ and long‐read technologies, the effort of Shi et al. allows the retrieval of a comprehensive collection of full‐length transcripts and their expression levels in both healthy and cancerous tissues. Specifically, a total of 43 *BRAF* transcript variants were reconstructed, most of which are *de novo* and noncoding. As mentioned above, *BRAF‐220* (*TCONS_00952581*) and *BRAF‐204* (*TCONS_00952571*) are both retrieved as full‐length and full‐splice variants.

To quantify *BRAF* transcript variants in additional cancer‐related RNA‐seq datasets, we focused on the 21 transcripts reported in Ensembl (release 113) because they are routinely updated within the GENCODE project, and we built an *ad hoc* bioinformatic pipeline called IsoWorm. IsoWorm (Fig. 1D) integrates two distinct methods: Salmon, a *state‐of‐the‐art* tool for transcript variant quantification, and a custom approach designed to specifically distinguish between the *BRAF‐220* and *BRAF‐204* variants. Salmon quantifies transcripts via a quasimapping approach and probabilistic modeling, which results in higher sensitivity compared to other methods [22]. Concerning the custom approach, we take advantage of two manually selected regions that are variant‐specific. In particular, the region distinctive for *BRAF‐220* corresponds to the most downstream ~ 1 kb out of the ~ 3.9 kb long 3'UTR, while the region distinctive for *BRAF‐204* corresponds to the most downstream ~ 4 kb out of the ~ 7 kb long 3'UTR (green boxes in Fig. S1). We then used custom GTF files encompassing the genomic coordinates of those regions. Following the read mapping step, the expression level of full‐length transcripts is estimated considering the library size of the dataset under study and the size of the selected regions. The advantages of IsoWorm include easy customization to quantify the transcript variants of interest, versatility in dealing with diverse input file formats from RNA‐seq experiments, acceptance of raw FASTQ files or alignment BAM files, and acquisition from the full experiment or a partial subset. Owing to the implementation of Salmon, the expression level of all the known transcripts of the gene of interest can be calculated, so that they can be ranked from the highest to the lowest expression. Owing to the implemented custom approach, IsoWorm estimates transcript expression from sliced BAM files or partial FASTQ files, favoring faster and lighter quantification of the transcripts of interest. However, transcripts are quantified in FPKM, which allows only intrasample comparisons [23]. IsoWorm is publicly available at https://github.com/ctglab/isoworm.

In conclusion, FLIBase and IsoWorm allow the correct identification and quantification of *BRAF* transcript variants in RNA‐seq datasets of human cancer.

### 3.3 *BRAF‐204* transcript variant is highly expressed across cancer cell lines and tissues

IsoWorm was used to quantify *BRAF* transcript variants in 690 CCLE cell lines belonging to 12 cancer types: central nervous system (C.N.S.), colorectal, hematopoietic and lymphoid, kidney, liver, lung, ovary, pancreas, prostate, skin, stomach, and thyroid.

**Fig. 2:**
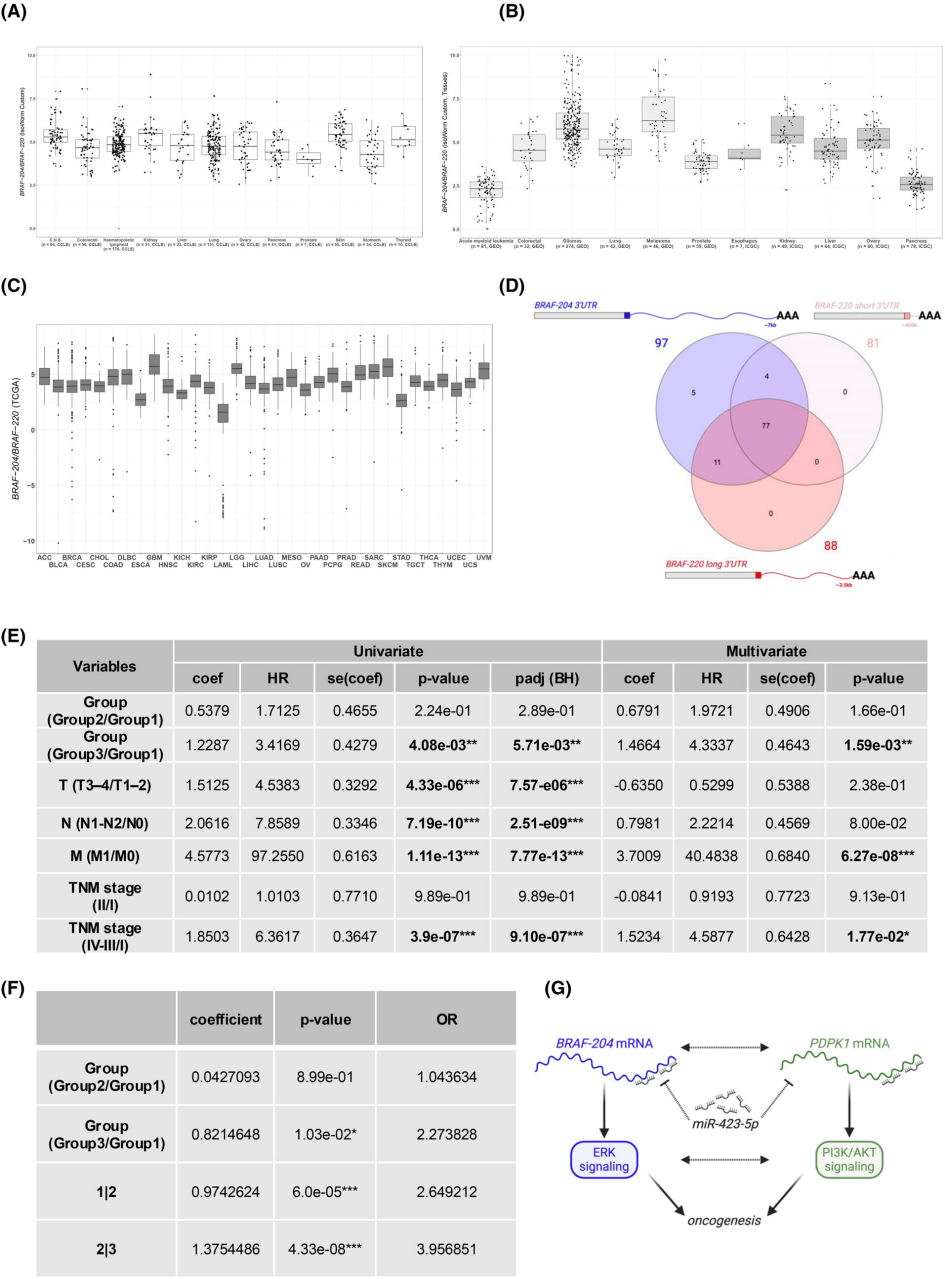
In cancer, the *BRAF‐204/BRAF‐220* ratio is consistently greater than 1 and is associated with patient survival. (A–C) Box plots of the *BRAF‐204/BRAF‐220* ratio. The horizontal line represents the median, the central box indicates the IQR, and the whiskers extend up to 1.5 times the IQR. (A, B) In (A) the results obtained from CCLE cell lines are presented (total *n* = 690, 12 cancer types). In (B) the results obtained from the GEO datasets are presented on the left (total *n* = 534, 6 cancer types), whereas the results obtained from the ICGC datasets are presented on the right (total *n* = 258, 5 cancer types). Quantification of *BRAF‐204* and *BRAF‐220* transcript variants and the calculation of their ratio were obtained using the custom module of IsoWorm. The ratios are expressed as log2(*BRAF‐204*_FPKM + 0.01/*BRAF‐220*_FPKM + 0.01). (C) The results obtained from the TCGA are presented (total *n* = 9219, 33 cancer types). The quantification of the *BRAF‐204* and *BRAF‐220* variants was obtained via FLIBase, and the *BRAF‐204/BRAF‐220* ratio was subsequently calculated. The ratios are expressed as log2(*BRAF‐204*_TPM + 0.01/*BRAF‐220*_TPM + 0.01). The ratios are consistently greater than 1 both in cancer cell lines and in tissue samples. (D) Venn diagram of lung cancer patients expressing *BRAF‐220* with a short 3'UTR (upstream polyA site located ~ 400 nt downstream of the stop codon, *light red*), *BRAF‐220* with a long 3'UTR (downstream polyA site located at the end of the ~ 3.9 kb 3'UTR, *dark red*), and *BRAF‐204* with an ~ 7 kb 3'UTR (terminal polyA site, *blue*). (E) Univariate (left) and multivariate (right) Cox proportional hazards regression analyses performed on 250 KIRP patients. The univariate model investigates how each factor (*BRAF‐204/BRAF‐220* ratio, size and extent of the primary tumor (T3–T4/T1–T2), involvement of regional lymph nodes (N1–N2/N0), presence of distant metastases (M1/M0), and tumor stage (TNM I, II, III–IV)) impacts patients' overall survival, whereas the multivariate model considers the impact of all factors simultaneously. Group 1 included patients with the lowest *BRAF‐204/BRAF‐220* ratios (≤ 33rd percentile (1Q)). Group 2 included patients with mid‐range *BRAF‐204/BRAF‐220* ratios (> 33rd percentile and ≤ 66th percentile (2Q)). Group 3 included patients with the highest *BRAF‐204/BRAF‐220* ratios (> 66th percentile (3Q)). T1–4: increasing size and extent of the primary tumor. N0/N1–N2: no evidence of a tumor in regional lymph nodes (N0) or evidence of a tumor in regional lymph nodes (N1–N2). M0/M1: no evidence of metastasis (M0) or evidence of metastasis (M1). TNM stage I–IV: progressive stage. Stages III and IV were merged. coef: the coefficient that quantifies the association between the independent variable and the log of the risk of the event. HR: the hazard ratio is the exponential of the coefficient. se(coef): the standard error of the coefficient measures the variability or uncertainty in the estimate of the coefficient. *P*‐value: calculated via the *z*‐test, the *P*‐value indicates the statistical significance of the coefficient. Adjusted *P*‐value (*P*adj): Benjamini–Hochberg (BH) correction for multiple comparisons. *< 0.05, **< 0.01, ***< 0.001. (F) Proportional odds logistic regression for ordered category outcome analysis was performed on 250 KIRP patients. The response variable was tumor stage (I < II < III–IV), while stage I was used as a reference. Group 1 included patients with the lowest *BRAF‐204/BRAF‐220* ratios (≤ 33rd percentile (1Q)). Group 2 included patients with mid‐range *BRAF‐204/BRAF‐220* ratios (> 33rd percentile and ≤ 66th percentile (2Q)). Group 3 included patients with the highest *BRAF‐204/BRAF‐220* ratios (> 66th percentile (3Q)). 1|2, 2|3: intercepts for the various levels of the outcomes. Coefficient: the coefficient represents the effect of an independent variable on the log odds of being in a higher category for the dependent variable. OR: odds ratio. *P*‐value: *< 0.05, ***< 0.001. (G) Working hypothesis to explain the association between high *BRAF‐204* levels and bad prognosis in KIRP. By sponging miR‐423, *BRAF‐204* might sustain *PDPK1* expression, so that both the ERK and the PI3K/AKT pathways are active.

We used the Salmon module to calculate the expression levels of all 21 *BRAF* transcript variants reported in Ensembl (release 113). The pie charts of variant expression illustrate that *BRAF‐204* is the most highly expressed variant in cancer cell lines, accounting for 53.6 ± 4.5% of all *BRAF* transcripts, whereas the *BRAF‐220* variant accounts for a much smaller fraction (2.5 ± 0.9%, Fig. 1E). As a direct consequence, *BRAF‐204* is 16 to 41 times more highly expressed than *BRAF‐220* (Fig. S2A). The expression levels of *BRAF‐220* and *BRAF‐204* were also calculated via the custom module of IsoWorm. We confirmed that the expression of *BRAF‐204* is 16–42 times greater than that of *BRAF‐220* (Fig. 2A). Overall, the *BRAF‐204/BRAF‐220* median ratios estimated by the two modules of IsoWorm were highly correlated (Spearman's correlation coefficient greater than 0.79 for all datasets, except for thyroid cell lines (*n* = 10), Fig. S2B). This attests to the robustness of the custom module of IsoWorm.

IsoWorm was also used to quantify *BRAF* transcript variants in 792 tissue samples belonging to 11 cancer types. They were obtained from GEO (acute myeloid leukemia, colorectal cancer, gliomas, lung cancer, melanoma, and prostate cancer) and ICGC (esophageal cancer, kidney cancer, liver cancer, ovarian cancer, and pancreatic cancer) databases.

The GEO datasets were analyzed via both quantification modules of IsoWorm. According to the Salmon module, *BRAF‐204* accounts for 53.3 ± 23% of all BRAF transcripts, whereas *BRAF‐220* accounts for a smaller fraction (3 ± 1.6%, Fig. 1F). In terms of the *BRAF‐204/BRAF‐220* ratio, the expression of *BRAF‐204* is 4 to 60 times greater than that of *BRAF‐220* (Fig. S3A). Similarly, the custom module of IsoWorm estimates that the expression of *BRAF‐204* is 5–75 times greater than that of *BRAF‐220* (Fig. 2B, left). The correlation between the calculations is very high (the Spearman's correlation coefficient of the median ratios is greater than 0.8 for all the datasets, Fig. S3B).

The ICGC datasets were analyzed only with the custom module of IsoWorm. This is because Salmon requires the size of the entire library and the expression level of each transcript, whereas for the ICGC datasets, we could access only the sliced BAM files containing reads from *BRAF* transcripts. Also, in this case, *BRAF‐204* is expressed at a level 6 to 30 times greater than that of *BRAF‐220* (Fig. 2B, right).

With FLIBase, we quantified *BRAF‐220* (*TCONS_00952581*) and *BRAF‐204* (*TCONS_00952571*) in tissue samples belonging to the 33 cancer types that compose the TCGA. Following the guidelines described in [24], we focused on primary tumor samples, for which the annotation of clinical variables is extensive and highly curated (total *n* = 9219, Table S3). The results reported in Fig. 2C highlight the widespread predominance of *BRAF‐204* over *BRAF‐220*, with a median ratio ranging between 1.5 and 45.

FLIBase allowed us to quantify *BRAF‐220*, *BRAF‐204*, and their ratio in normal tissues as well. We analyzed 2599 samples belonging to 22 different tissues at GTEx (Table S4), and we observed that the higher expression levels of *BRAF‐204* over *BRAF‐220* characterize normal tissues too (the median ratio ranges between 9 and 37, Fig. S4A). Interestingly, the box plots of *BRAF‐220* and *BRAF‐204* expression levels in both TCGA (cancer, Fig. S5A) and GTEx (normal, Fig. S4B) tissue samples are in agreement with the pie charts in Fig. 1E,F, and they cumulatively indicate that *BRAF‐220*/*BRAF‐204* ratios are dictated by the expression level of the *BRAF‐204* variant (in *blue*), while *BRAF‐220* levels, besides being much lower, show less variability across tissues (in *red*).

We also quantified total *BRAF* levels. The results are reported in Fig. S2C for the CCLE cell lines, Fig. S3C for the GEO tissue samples, Fig. S5B for the TCGA tissue samples, and Fig. S4C for the GTEx tissue samples. The *BRAF‐204*/*BRAF‐220* ratio does not correlate with the total *BRAF* level in any of the datasets.

We highlight the extreme case of AML, which is characterized by the lowest *BRAF‐204/BRAF‐220* ratio (Fig. 2B,C). This is due to multiple factors, including low expression of *BRAF‐204*, high expression of *BRAF‐220*, and high expression of additional transcript variants (Fig. 1F and Fig. S5A). AML even shows samples that are devoid of *BRAF‐204* expression (3/81 (4%) in the GEO dataset and 24/150 (16%) in the TCGA dataset). Furthermore, AML is characterized by the highest level of total *BRAF* (Figs S3C and S5B). Interestingly, these features are shared across other leukemia types such as ALL, CLL, and CML (Fig. S6). Since *BRAF* mutations have been reported in AML and CLL and are associated with poor prognosis [25, 26, 27], our findings contribute to light up the interest of the scientific community on *BRAF* gene in the context of these leukemia subtypes. As soon as RNA‐seq datasets become available, it will be interesting to study *BRAF‐204*, *BRAF‐220*, and their ratio also in the context of HCL, a leukemia subtype where the BRAFV600E mutation is present in virtually all cases, it is well established as a driver, and it is extensively used as a target [28].

The new analysis of the *BRAF‐204/BRAF‐220* ratio in TCGA samples slightly differs from our previous analysis [5], a discrepancy that is ascribable to both biological and technical factors.

At the biological level, we note that the TCGA dataset is constantly curated, which means that the number, identity, stage, and/or subtype of current tissue samples is not the same as those in 2017.

At the technical level, several factors must be considered. First, the strategy for transcript variant quantification from short‐read data inevitably relies on the bulk of variants that are present in repositories at the time of analysis. In reference [5], we considered the 15 variants that were present in Ensembl (release 80) plus NCBI (GRCh38.p1). Then, we dismissed most of them based on the absence of transcription (no reads on variant‐specific exons or exon–exon junctions). Finally, we established that transcription across the splicing site located in exon 18 was distinctive between the *ref* variant and the *X1*/*X2* variants. Therefore, we quantified *BRAF‐ref* by counting the reads that spanned this splicing site. However, among the *BRAF* variants that compose current databases, many share the same splicing site and show some degree of transcription. For example, among the 21 *BRAF* variants that are currently present in Ensembl (release 113, Fig. S1A), 10 share that splicing site with *BRAF‐220* and are transcribed (see Fig. 1E,F and Fig. S6A). Therefore, we conclude that in [5] *BRAF‐ref* levels might have been overestimated. Conversely, we note that the quantification through IsoWorm that we describe in this article might suffer from the opposite bias, that is, underestimation of *BRAF‐220* levels. As mentioned above, the current annotation of *ENST00000646891.2/BRAF‐220* presents a much longer 3'UTR than the one we considered in 2017 (~ 100 nt vs. ~ 3.9 kb). The longer 3'UTR allowed us to select a variant‐specific region for *BRAF‐220* identification and quantification (most downstream ~ 1 kb). However, this comes at the price of underestimation of expression levels if there are no reads that map to the UTR until the very 3′ end. To address this issue, we resorted to a publicly available dataset in which a QuantSeq 3' mRNA library was built to produce fragments close to the 3′ end of polyA sites on RNA extracted from 97 lung cancer patients [13]. Specifically, we inferred the sizes of the *BRAF‐220* and *BRAF‐204* 3'UTRs by retrieving the positions of the polyA sites. According to our analysis, the *BRAF‐204* 3'UTR contains 1 polyA site, which is present in all 97 samples and is located at the end of ~ 7 kb (chr7: 140726516–140719327) (Fig. 2D, in blue). This experimental evidence matches our previous findings [5] and confirms that *BRAF‐204* mRNA is consistently expressed with a very long, homogeneously transcribed 3'UTR. Conversely, the *BRAF‐220* 3'UTR contains two polyA sites. The first one is located ~ 400 nt downstream of the stop codon (chr7:140734531–140734607), and it is present in 81 out of 97 samples. The second one is located ~ 3.6 kb downstream of the stop codon (chr7:140731035–140731126), and it is present in 88 out of 97 samples. Interestingly, the short and long 3'UTRs are coexpressed in most samples (77 out of 97) (Fig. 2D, in red). These results indicate that the long version of the *BRAF‐220* 3'UTR is indeed expressed and should be selected for normalization of variant levels. However, these and previous analyses [5] also indicate that the size of the *BRAF‐204* 3'UTR is highly variable, both within and across samples. Therefore, the choice of the long 3'UTR is inherently linked with a certain degree of underestimation.

In addition, the precomputed quantification of *BRAF‐204* and *BRAF‐220* produced through FLIBase on TCGA tissue samples combines the results of short‐ and long‐read sequencing, whereas the quantification we performed in [5] was uniquely based on short reads.

In conclusion, precise quantification of transcript variants via RNA‐seq remains problematic despite the efforts of the bioinformatics community in developing increasingly sophisticated methods. The use of short‐read and bulk sequencing presents inherent technical limitations and results in very divergent estimations [10]. Nonetheless, the technical and biological aspects described above prompt us to consider the quantification of *BRAF* variants presented here (*BRAF‐204* is the predominant transcript variant in human cancer) as more accurate than the quantification presented in [5].

### 3.4 The *BRAF‐204*/*BRAF‐220* ratio has prognostic value in KIRP

We assessed whether the *BRAF‐204/BRAF‐220* ratio has an impact on patient prognosis via the quantification of *TCONS_00952571/BRAF‐204* and *TCONS_00952581/BRAF‐220* in TCGA samples, as reported in FLIBase. We selected 11 cancer types that are characterized by the highest frequency of *BRAF* alterations [16]. Five of them present *BRAF* mutations in more than 4% of the samples (COAD, LUAD, SKCM, THCA, and UCEC), whereas six present *BRAF* copy number variations in more than 60% of the samples (ACC, ESCA, GBM, KIRP, OV, and TGCT). For each of these 11 cancer types, we evaluated the association between the *BRAF‐204*/*BRAF‐220* ratio and patient prognosis (Table S1).

According to univariate Cox proportional hazards regression analysis, the *BRAF‐204/BRAF‐220* ratio impacts overall survival in KIRP‐*kidney renal papillary cell carcinoma* patients. Specifically, 250 patients were analyzed (Table S5), and patients in Group 3 (highest ratios, > 66th percentile (3Q)) have an increased risk of worse outcomes compared to patients in Group 1  (lowest ratios, ≤ 33rd percentile (1Q)). As reported in Table S1 and Fig. 2E, left, the hazard ratio (HR) is > 3.

We then calculated the HR for prognostic factors that are already known to increase the risk of worse outcomes in KIRP patients, namely, the size and extent of the primary tumor (T3‐T4/T1‐T2), the involvement of regional lymph nodes (N1–N2/N0), the presence of distant metastases (M1/M0), and the overall tumor stage (TNM I, II, III–IV) [29]. As expected, we obtained HR values higher than 1 for all of them (Fig. 2E, left).

Next, we performed a multivariate Cox proportional hazards regression analysis, considering all the above‐mentioned prognostic variables at the same time. As shown in Fig. 2E, right, a high *BRAF‐204/BRAF‐220* ratio is still associated with poor survival (HR > 4). Therefore, we can conclude that in KIRP the ratio of *BRAF* variants is an independent prognostic factor of worse outcome.

In addition, we assessed whether having a higher *BRAF‐204/BRAF‐220* ratio increases the probability of being in a higher KIRP stage. To this end, we performed a proportional odds logistic regression (POLR) for ordered category outcome analysis. We used stage I as a reference and considered tumor stage as the response variable (I < II < III–IV). Consistent with the hazard regression analyses, we obtained an odds ratio of 2.27 for Group 3, which means that KIRP patients with the highest ratios have higher odds of having a more advanced tumor stage than patients with the lowest ratios (Fig. 2F).

Finally, since KIRP is one of the six cancer types with *BRAF* copy number variations in more than 60% of samples, we tested whether there is a correlation between copy number status (i.e., 0, 1, 2, 3) and the *BRAF‐204/BRAF‐220* ratio, but we did not find it (Spearman's rho −0.09, *P* < 0.14).

To date, several molecular biomarkers have been proposed to improve the prediction of drug responsiveness and disease outcome in KIRP (see, for example, [30, 31, 32, 33, 34]), but the role of *BRAF* has just begun to be explored [16]. Crucially, our findings reveal that high *BRAF‐204/BRAF‐220* ratios distinguish patients with worse outcomes. They also spur to fill a gap in public repositories, since we lack an independent KIRP cohort suitable for validating the prognostic value of proposed biomarkers.

As mentioned in the Introduction, we have previously identified 20 microRNAs that bind to *BRAF‐204* 3'UTR, modulating its expression and, consequently, the output of the ERK signaling pathway [7]. These miRNAs are variant‐specific, that is, they are different from those that target *BRAF‐220* 3'UTR (see, e.g., the list of experimentally validated miRNA reported in https://awi.cuhk.edu.cn/~miRTarBase/miRTarBase_2025/php/index.php). This indicates that the two transcripts are involved in distinct miRNA‐centered regulatory networks. To formulate a hypothesis that might explain the association between high *BRAF‐204* levels and bad prognosis in KIRP, we investigated whether *BRAF‐204* works as ceRNA, that is, whether it sustains the expression of another oncogene by sponging an oncosuppressive miRNA [35]. Thanks to the availability of the miRNome at TCGA, we performed a rank correlation analysis between the *BRAF‐204* transcript and KIRP miRNAs. Then, we focused on the previously identified *BRAF‐204*‐targeting miRNAs. Among those expressed in KIRP, we found two that present a significant negative correlation with *BRAF‐204*: miR‐3651 and miR‐423‐5p (Table S6 and Fig. S7A,B). We also performed a rank correlation analysis between the *BRAF‐204* transcript and the other KIRP transcripts. Then, we focused on the experimentally validated targets of miR‐423 that are listed in MiRTarBase [36] (aside from *BRAF‐204*, no other experimentally validated targets are currently reported for miR‐3651). Among miR‐423 targets expressed in KIRP, we found that *ENSG00000140992*/*PDPK1* [37] presents a significant positive correlation with *BRAF‐204* and a significant negative correlation with miR‐423 (Table S7 and Fig. S7C,D). miR‐423 is a well‐studied tumor suppressive miRNA involved in several ceRNA networks [37, 38, 39, 40, 41, 42, 43]. On the other hand, PDPK1 is one of the most crucial kinases of the highly oncogenic PI3K/AKT pathway, as it is responsible for AKT activation by phosphorylation at Thr308 [44]. Therefore, this ceRNA network suggests that *BRAF‐204* and *PDPK1* promote oncogenesis by sponging miR‐423. It is worth mentioning that, besides providing a working hypothesis for further investigations on the role played by BRAF‐204, PDPK1, and miR‐423 in KIRP, this regulatory network entails that the intricate cross‐talk between the ERK and the PI3K/AKT oncogenic pathways [45] does not involve only proteins and might extend to transcripts (Fig. 2G).

Contrary to KIRP, we found no impact of the *BRAF‐204/BRAF‐220* ratio on THCA or SKCM, that is, the cancer types characterized by the highest percentage of patients carrying *BRAF* mutations. However, given the availability of larger datasets, it might be interesting to assess whether the ratio has prognostic value in the BRAFV600E‐negative subgroup, whereas the presence of the mutant protein takes over in the BRAFV600E‐positive subgroup.

In more general terms, the results we obtained in KIRP underscore the importance of going beyond the study of the aberrant activity of the mutant kinase and of including the study of the expression of the *BRAF* gene.

## 4 Conclusions

Quantifying full‐length transcript variants from sequencing data poses significant challenges from both technical and biological points of view [10]. The case of *BRAF* is particularly complex because the variants reported in databases are many, very similar, and rapidly changing, and because their length varies within and across samples due to the use of alternative polyA sites. To tackle this issue, we built the bioinformatic pipeline IsoWorm, which implements two methods for variant identification and quantification. IsoWorm was used to quantify *BRAF* transcripts in 690 cell lines belonging to 12 cancer types and 1020 tissue samples belonging to 14 cancer types. Thanks to FLIBase, we also reanalyzed TCGA data, which included 9219 tissue samples belonging to 33 cancer types. Through the combined use of multiple computational approaches, we establish that in human cancer *BRAF* is a mix of two full‐length, protein‐coding, and biologically relevant isoforms, namely, *BRAF‐220* and *BRAF‐204*. Crucially, *BRAF‐204* mRNA is predominant over *BRAF‐220* mRNA, and in solid cancers, it also stands as the most expressed among all *BRAF* transcript variants. Conversely, leukemia samples are characterized by a variegated expression of multiple *BRAF* transcript variants. The imbalance against *BRAF‐220* is much less pronounced, and a fraction of samples are devoid of *BRAF‐204*.

We also show that the *BRAF‐204*/*BRAF‐220* ratio impacts patient survival. Specifically, in KIRP, the prognosis of patients with extreme *BRAF‐204*/*BRAF‐220* ratios is worse. Although the study of *BRAF* and its transcript variants in this cancer type is still in its infancy, we propose that the *BRAF‐204* transcript might sustain not only the ERK pathway but also the PI3K/AKT pathway by sponging the oncosuppressive miR‐423 and relieving its targeting of *PDPK1*.

Historically, BRAF‐220 has been considered the only BRAF isoform and has represented the undivided focus of *in vitro* and *in vivo* model systems [46, 47]. Conversely, the *in silico* analyses presented here, together with the experimental characterization described by us and others in [4, 5, 6, 7, 8, 9, 17, 18, 19, 48], introduce a paradigm shift in BRAF‐centered studies [49], prompting us to consider BRAF‐204 as ‘the real’ BRAF. They also provide the rationale for benchmarking BRAF‐204 against BRAF‐220. Similarities and differences should be systematically investigated at multiple levels. From a basic research point of view, we must deepen our knowledge of the mechanisms that regulate isoform expression and functioning. In addition, translational research can benefit from the analysis of the two isoforms in terms of sensitivity and intrinsic/acquired resistance to drugs. Besides the targeting of the mutant BRAFV600E kinase and the aberrantly active ERK pathway, we should evaluate whether it is beneficial to concomitantly target kinase‐independent and/or coding‐independent activities that BRAF‐204 and BRAF‐220 isoforms might have. Finally, the *BRAF‐204*/*BRAF‐220* ratio can support clinicians in the choice of therapeutic regimens and the management of relapse [50].

## Conflict of interest

The authors declare no conflict of interest.

## Author contributions

RDA and LP conceived the project. MSP, RDA, and LP designed the analyses. MSP, DT, and GM analyzed the data. AM, RDA, and LP supervised the research. All the authors wrote, discussed, and approved the manuscript.

## Peer review

The peer review history for this article is available at https://www.webofscience.com/api/gateway/wos/peer‐review/10.1002/1878‐0261.70043.

## Supporting information


**Fig. S1.** Related to Fig. 1. The 21 *BRAF* transcripts deposited in Ensembl (release 113) and variant‐specific regions used by the custom module of IsoWorm for *BRAF‐204* and *BRAF‐220* quantification.
**Fig. S2.** Related to Fig. 2. *BRAF‐204/BRAF‐220* ratio and total *BRAF* levels in CCLE cell lines (*n* = 690, 12 cancer types).
**Fig. S3.** Related to Fig. 2. *BRAF‐204/BRAF‐220* ratio and total *BRAF* levels in GEO tissue samples (*n* = 534, 6 cancer types).
**Fig. S4.** Related to Fig. 2. Box plots of *BRAF‐204/BRAF‐220* ratio, *BRAF‐220*, *BRAF‐204*, and total *BRAF* levels in the normal tissue samples that compose the GTEx (*n* = 2599 samples, 22 normal tissues).
**Fig. S5.** Related to Fig. 2. Box plots of *BRAF‐220*, *BRAF‐204*, and total *BRAF* levels in the cancer tissue samples that compose the TCGA (*n* = 9219 samples, 33 cancer types).
**Fig. S6.** Related to Fig. 2. *BRAF‐204* transcript variant is highly expressed across 4 leukemia types.
**Fig. S7.** Related to Fig. 2. Spearman correlation of *BRAF‐204* with miR‐3651, miR‐423, and *PDPK1* in the KIRP dataset at TCGA (*n* = 290).

**Table S1.** Univariate Cox proportional hazards regression on 11 TCGA cancer types.

**Table S2.** Brant Wald test on the proportional odds logistic regression for ordered category outcomes analysis performed on 250 KIRP patients.

**Table S3.** TCGA cancer tissue samples (primary tumors).

**Table S4.** GTEx normal tissue samples.

**Table S5.** Baseline characteristics of the 250 KIRP patients used for hazards regression analyses.

**Table S6.** Correlation of *BRAF‐204* with targeting miRNAs that are expressed in the KIRP dataset at TCGA.

**Table S7.** Correlation of *BRAF‐204* and miR‐423 with the experimentally validated targets of the miRNA that are expressed in the KIRP dataset at TCGA.

## Data Availability

The tools and datasets used during the current study are as follows: Tools: (1) ffq: https://github.com/pachterlab/ffq. (2) Salmon: https://github.com/COMBINE‐lab/Salmon. (3) SAMtools: https://www.htslib.org/. (4) Snakemake: https://snakemake.github.io/. (5) STAR: https://github.com/alexdobin/STAR. (6) Stringtie: https://ccb.jhu.edu/software/stringtie/. Datasets: (1) RNA‐seq data of cancer cell lines were retrieved from the Cancer Cell Line Encyclopedia (CCLE, https://sites.broadinstitute.org/ccle/) under the SRA ID SRP186687. (2) RNA‐seq data of cancer tissue samples were retrieved from the Gene Expression Omnibus (GEO, https://www.ncbi.nlm.nih.gov/gds; ID GSE181157 (ALL), GSE63646 (AML), GSE161711 (CLL), GSE100026 and GSE144119 (CML), GSE146009 (colorectal), GSE48865 (gliomas), GSE140343 (lung), GSE91061 (melanoma), and GSE138503 (prostate)); the International Cancer Genome Consortium (ICGC, https://dcc.icgc.org/); The Cancer Genome Atlas (TCGA, https://www.cancer.gov/ccg/research/genome‐sequencing/tcga). (3) QuantSeq 3' mRNA‐Seq REV FASTQ data and metadata from lung cancer tissue samples were retrieved from GEO (ID GSE174330). All the data generated during the current study are available from the corresponding authors upon reasonable request. The IsoWorm pipeline is available at https://github.com/ctglab/isoworm.
