# Supplementary material for: Landscape of BRAF transcript variants in human cancer
Source: Mol Oncol. 2025 May 25;19(9):2700–14. doi: 10.1002/1878-0261.70043 (PMC12420348; doi:10.1002/1878-0261.70043)
Supplement: Supplementary file 1 — Fig. S1. Related to Fig. 1. The 21 BRAF transcripts deposited in Ensembl (release 113) and variant‐specific regions used by the custom module of IsoWorm for BRAF‐204 and BRAF‐220 quantification. Fig. S2. Related to Fig. 2. BRAF‐204/BRAF‐220 ratio and total BRAF levels in CCLE cell lines (n = 690, 12 cancer types). Fig. S3. Related to Fig. 2. BRAF‐204/BRAF‐220 ratio and total BRAF levels in GEO tissue samples (n = 534, 6 cancer types). Fig. S4. Related to Fig. 2. Box plots of BRAF‐204/BRAF‐220 ratio, BRAF‐220, BRAF‐204, and total BRAF levels in the normal tissue samples that compose the GTEx (n = 2599 samples, 22 normal tissues). Fig. S5. Related to Fig. 2. Box plots of BRAF‐220, BRAF‐204, and total BRAF levels in the cancer tissue samples that compose the TCGA (n = 9219 samples, 33 cancer types). Fig. S6. Related to Fig. 2. BRAF‐204 transcript variant is highly expressed across 4 leukemia types. Fig. S7. Related to Fig. 2. Spearman correlation of BRAF‐204 with miR‐3651, miR‐423, and PDPK1 in the KIRP dataset at TCGA (n = 290). [file MOL2-19-2700-s005.pdf]

a)

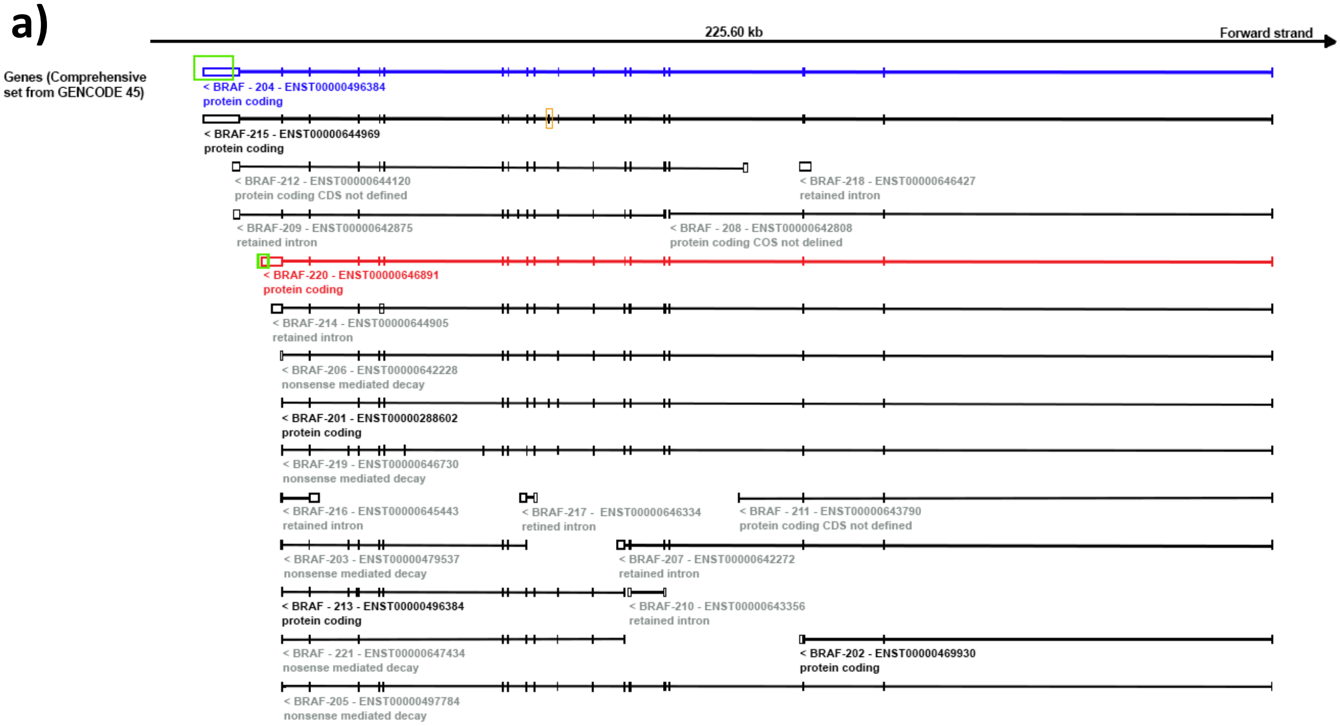

b)

QUERY	SCORE	START	END	QSIZE	IDENTITY	CHROM	STRAND	START	END	SPAN
BRAF-204	5819	1	5819	5819	100.0%	chr7	-	140719327	140725145	5819
BRAF-204	233	11	299	5819	92.4%	chr1	+	103095627	103095927	301
BRAF-204	28	1952	1987	5819	75.0%	chr1	-	85993060	85993091	32

c)

QUERY	SCORE	START	END	QSIZE	IDENTITY	CHROM	STRAND	START	END	SPAN
BRAF-220	1900	1	1900	1900	100.0%	chr7	-	140730665	140732564	1900
BRAF-220	332	1222	1580	1900	96.5%	chr3	-	90303793	90304127	335
BRAF-220	331	1226	1580	1900	99.5%	chr11	+	119270240	119270745	506

Supplementary Figure 1-related to Figure 1. The 21 *BRAF* transcripts deposited in Ensembl (release 113) and variant-specific regions used by the custom module of IsoWorm for *BRAF-204* and *BRAF-220* quantification.
BRAF gene is located on chromosome 7 and is transcribed from the reverse strand.
a) In Ensemble (113) 5 *BRAF* transcript variants are deposited as protein-coding, 3 as protein-coding with CDS not defined, 7 as containing retained introns, and 6 as undergoing nonsense-mediated decay.
BRAF-204 is highlighted in blue, while *BRAF-220* is highlighted in red. For the quantification of these two variants, the custom module of IsoWorm uses the variant-specific regions boxed in green.
Of note, there is only one exon that distinguishes *BRAF-204* from *BRAF-215* (yellow box here, and yellow slice in the pie charts reported in **Fig.1e-f** and **Supplementary Fig. 6a**).
b-c) Top 3 Blat alignment between the variant-specific regions boxed in green in panel **a** and the whole reference genome (GRCh38.p14). The highlighted rows show a much higher score and a full match on chromosome 7 for both the *BRAF-204*-specific region (**b**) and the *BRAF-220*-specific region (**c**).

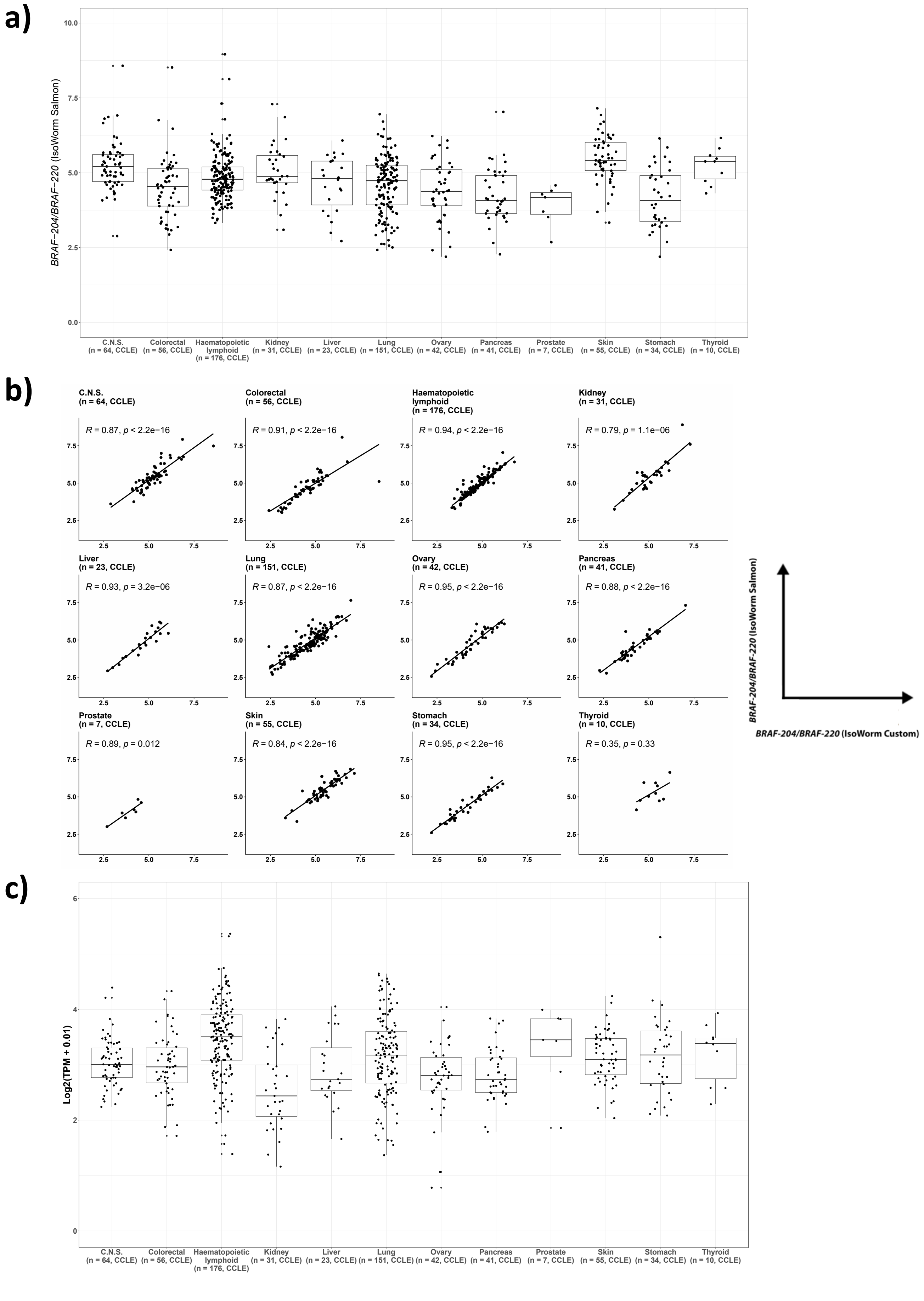

Supplementary Figure 2-related to Figure 2. *BRAF-204/BRAF-220* ratio and total *BRAF* levels in CCLE cell lines (n = 690, 12 cancer types).
a) Box plots of *BRAF-204/BRAF-220* ratio. Quantification of *BRAF-204* and *BRAF-220* variants, followed by the calculation of their ratio, was made using the Salmon module of IsoWorm. Ratios are expressed as log2(*BRAF-204*_TPM + 0.01/*BRAF-220*_TPM + 0.01). They are consistently greater than 1.
b) Spearman test to assess the correlation between the quantification of *BRAF-204/BRAF-220* ratio obtained with the Salmon module (y axis, **panel a**) and the custom module (x axis, **Fig. 2a**) of IsoWorm.
c) Box plots of total *BRAF* levels, calculated using the Salmon module of IsoWorm and the trixmeta R package, and expressed as log2(TPM + 0.01).
In the box plots, the horizontal line represents the median, the central box indicates the IQR, and the whiskers extend up to 1.5 times the IQR.

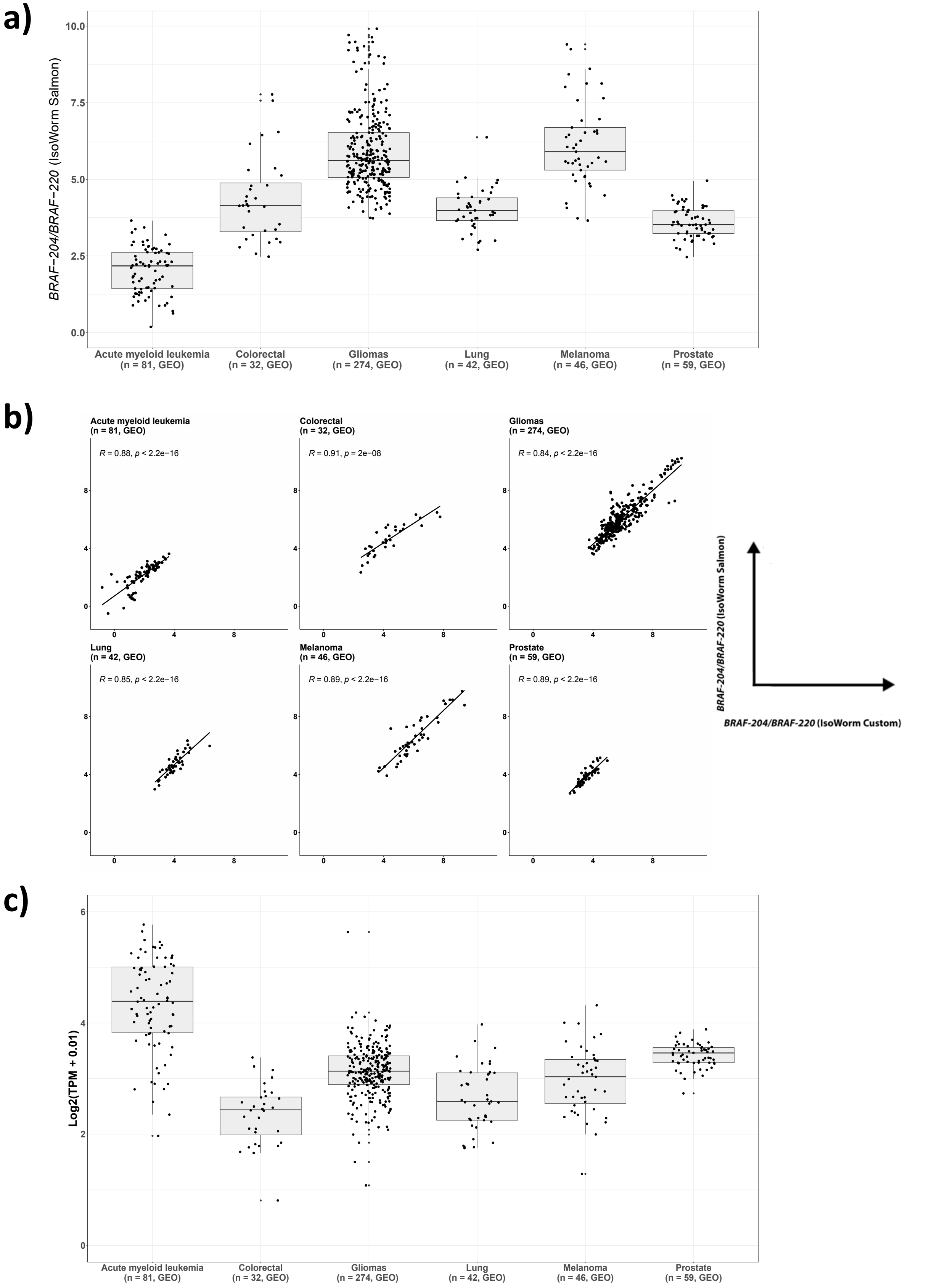

Supplementary Figure 3-related to Figure 2. *BRAF-204/BRAF-220* ratio and total *BRAF* levels in GEO tissue samples (n = 534, 6 cancer types).

a) Box plots of *BRAF-204/BRAF-220* ratio. Quantification of *BRAF-204* and *BRAF-220* variants, followed by the calculation of their ratio, was made using the Salmon module of IsoWorm. Ratios are expressed as log2(*BRAF-204*_TPM + 0.01/*BRAF-220*_TPM + 0.01). They are consistently greater than 1.

b) Spearman test to assess the correlation between the quantification of *BRAF-204/BRAF-220* ratio obtained with the Salmon module (y axis, **panel a**) and the custom module (x axis, **Fig. 2b**) of IsoWorm.

c) Box plots of total *BRAF* levels, calculated using the Salmon module of IsoWorm and the trixmeta R package, and expressed as log2(TPM + 0.01).

In the box plots, the horizontal line represents the median, the central box indicates the IQR, and the whiskers extend up to 1.5 times the IQR.

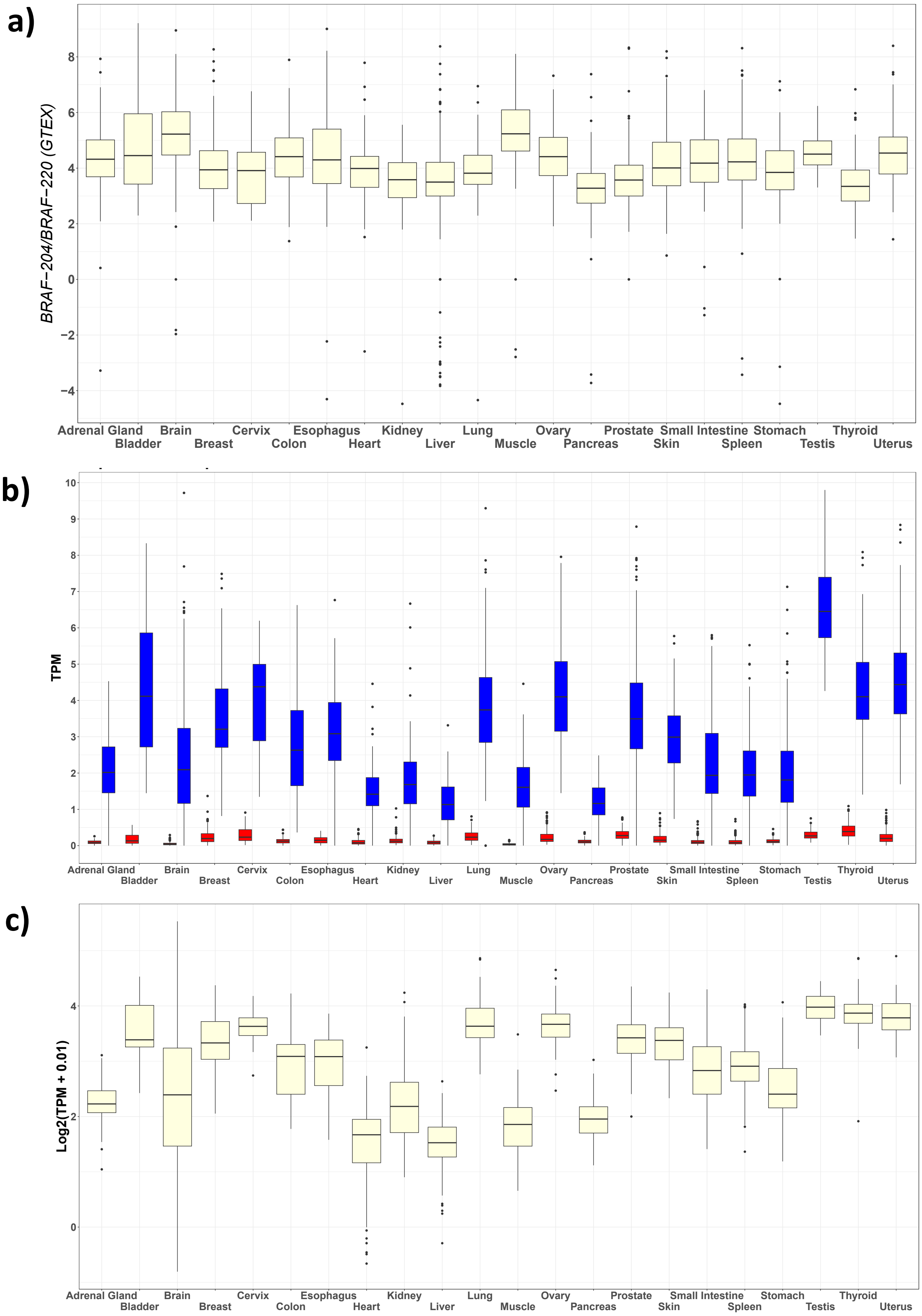

Supplementary Figure 4-related to Figure 2. Box plots of *BRAF-204/BRAF-220* ratio, *BRAF-220*, *BRAF-204,* and total *BRAF* levels in the normal tissue samples that compose the GTEx (n = 2599 samples, 22 normal tissues).
a-b) The quantification of the *BRAF-220* (red) and *BRAF-204* (blue) variants in TPM was obtained via FLIBase. The *BRAF-204/BRAF-220* ratios were subsequently calculated as log2(*BRAF-204*_TPM + 0.01/*BRAF-220*_TPM + 0.01). Ratios are shown in **a** and are consistently greater than 1, while *BRAF-204* and *BRAF-220* levels are shown in **b**.
c) Total *BRAF* levels were calculated using the recount3 package in R. They are expressed as log2(TPM + 0.01).
The horizontal line represents the median, the central box indicates the IQR, and the whiskers extend up to 1.5 times the IQR.

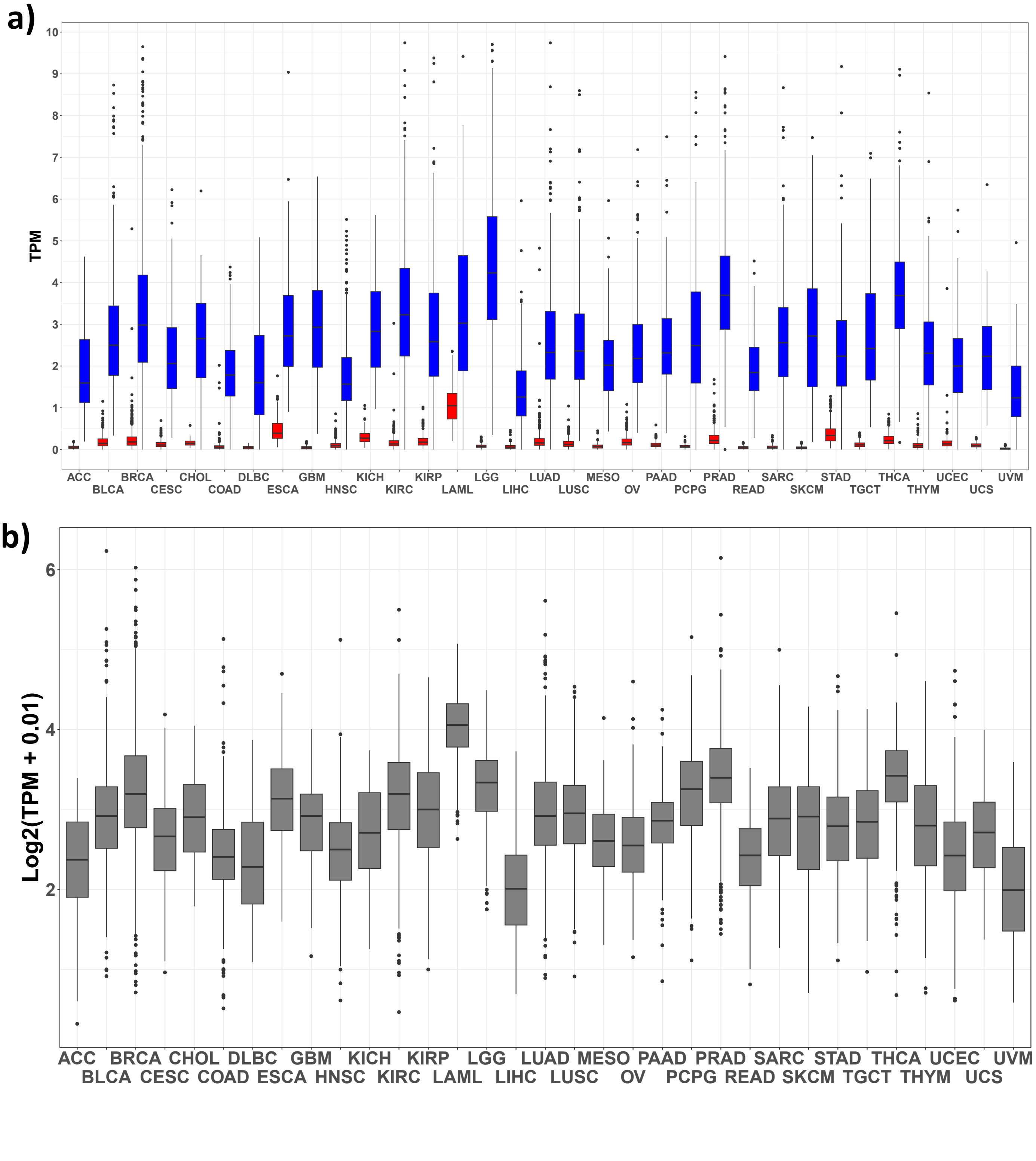

Supplementary Figure 5-related to Figure 2. Box plots of *BRAF-220*, *BRAF-204,* and total *BRAF* levels in the cancer tissue samples that compose the TCGA (n = 9219 samples, 33 cancer types).

a) The quantification the *BRAF-220* (red) and *BRAF-204* (blue) variants in TPM was obtained via FLIBase. The *BRAF-204/BRAF-220* ratios were subsequently calculated and are shown in **Fig. 2c**.

b) Total *BRAF* levels were calculated using the recount3 package in R. They are expressed as log2(TPM + 0.01).

The horizontal line represents the median, the central box indicates the IQR, and the whiskers extend up to 1.5 times the IQR.

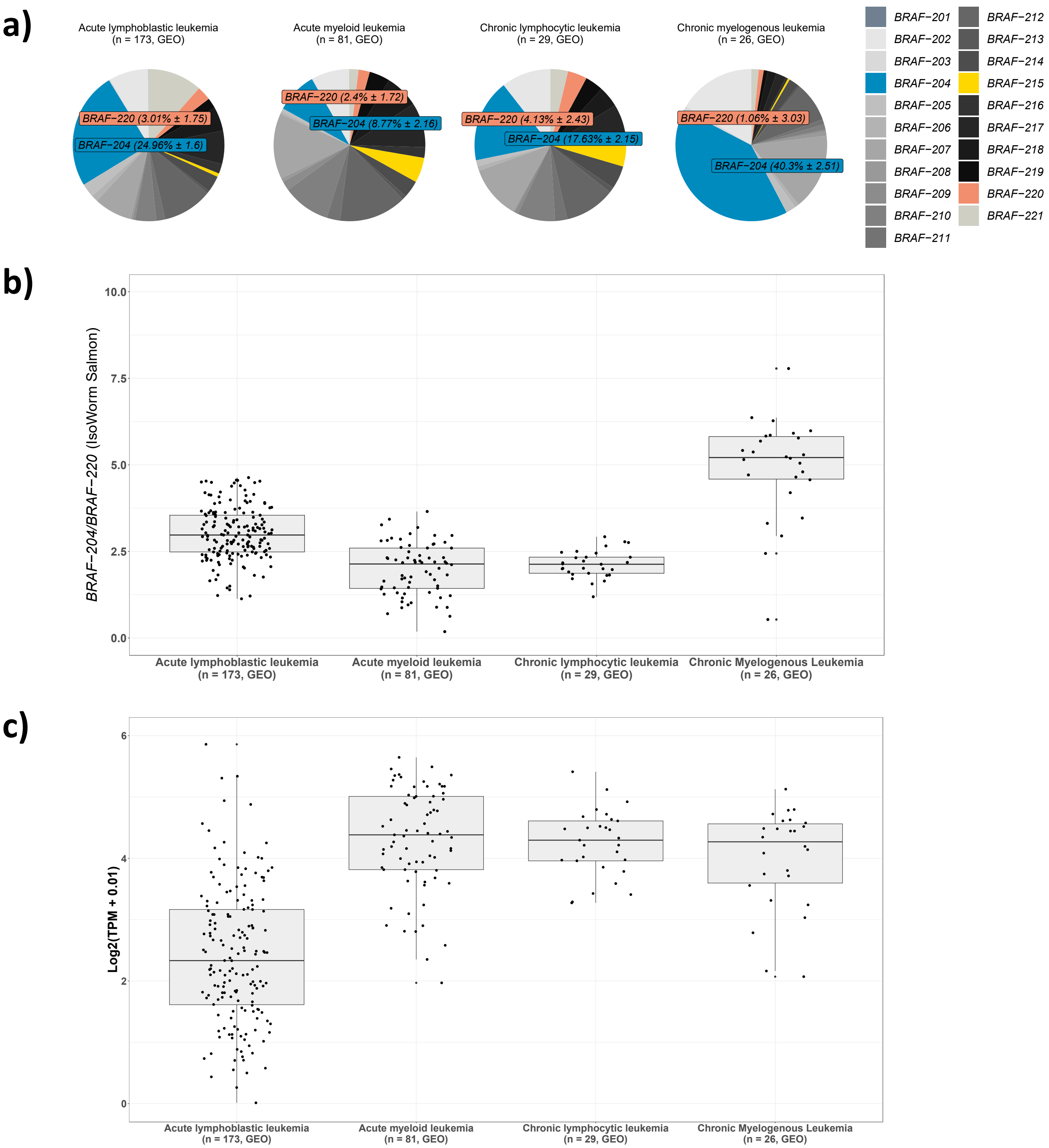

Supplementary Figure 6-related to Figure 2. *BRAF-204* transcript variant is highly expressed across 4 leukemia types.
a) Pie charts showing the 21 transcript variants of *BRAF* present in Ensembl (113). Their mean relative abundance (%) was estimated via the Salmon module of IsoWorm. *BRAF-204* is highlighted in *blue*, whereas *BRAF-220* is highlighted in *red*. Mean±SD values are indicated.
a) Box plots of *BRAF-204/BRAF-220* ratio. Quantification of *BRAF-204* and *BRAF-220* variants, followed by the calculation of their ratio, was made using the Salmon module of IsoWorm. Ratios are expressed as log2(*BRAF-204*_TPM + 0.01/*BRAF-220*_TPM + 0.01). They are consistently greater than 1.
c) Box plots of total *BRAF* levels, calculated using the Salmon module of IsoWorm and the trixmeta R package, and expressed as log2(TPM + 0.01).
In the box plots, the horizontal line represents the median, the central box indicates the IQR, and the whiskers extend up to 1.5 times the IQR.
Like AML samples, CLL samples are characterized by low *BRAF-204/BRAF-220* ratio (<2), while CLL and CML samples are characterized by high total *BRAF* levels (>4). In addition, all four leukemia types show a variegated expression of multiple *BRAF* transcript variants. For AML and CLL, we highlight the *BRAF-215* transcript variant (*yellow*), which is identical to *BRAF-204*, except for the presence of an extra exon between the 9th and the 10th exon (see **Supplementary Fig. 1**). If experimentally confirmed, the translation of this exon would result in 41 extra amino acids between the CR2 domain and the kinase domain.

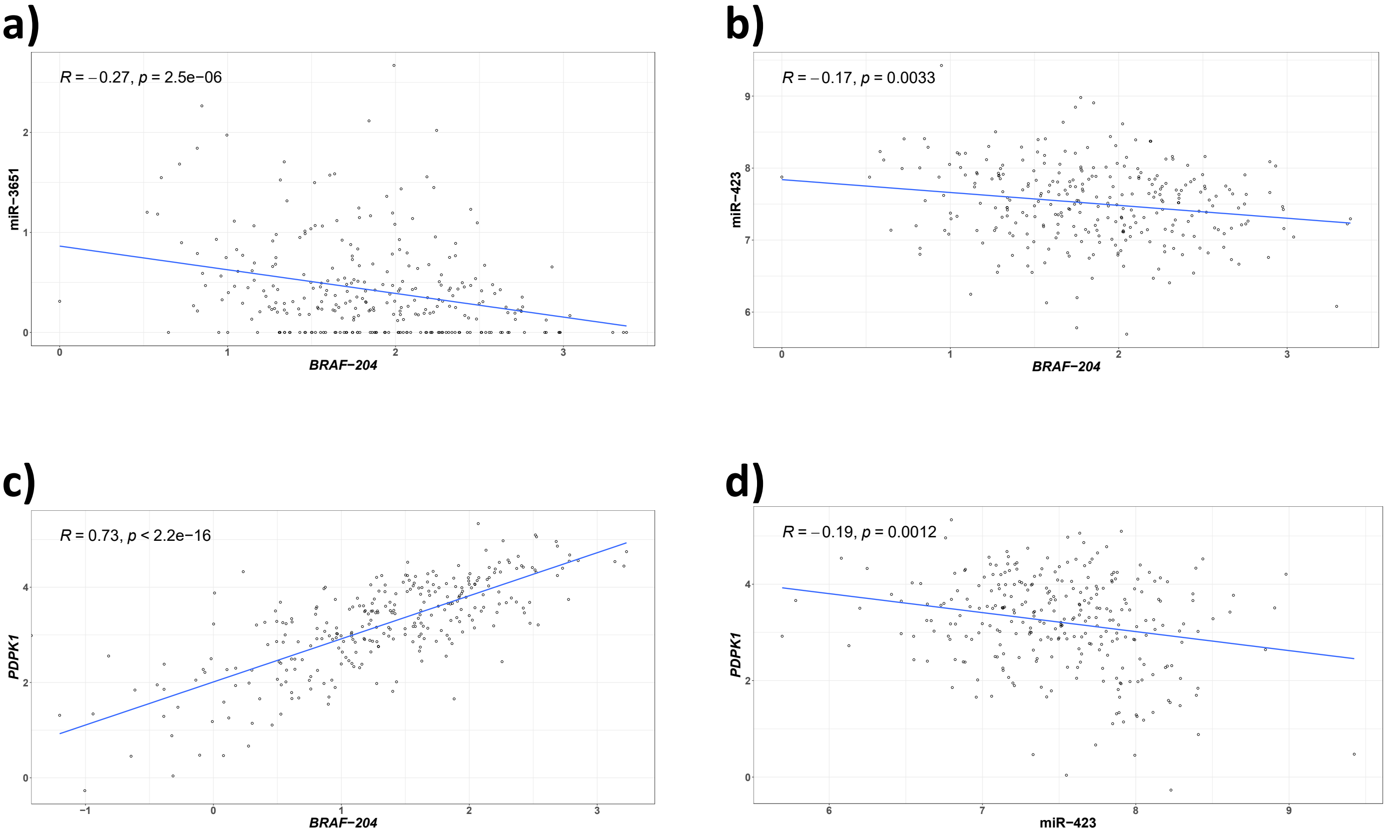

Supplementary Figure 7-related to Figure 2. Spearman correlation of *BRAF-204* with miR-3651, miR-423, and *PDPK1* in the KIRP dataset at TCGA (n = 290).

The quantification of the *BRAF-204* transcript was retrieved from the FLIBase database. The quantification of *PDPK1* was obtained directly from the TCGA dataset using the recount3 library in R. The quantification of miR-3651 and miR-423 was obtained from the GDC TCGA project. Transcript expression levels are expressed as log2(TPM+1), while miRNA expression levels are expressed as log2(RPM+1).

BRAF-204 negatively correlates with both miR-3651 and miR-423 (**a**, **b**) and positively correlates with *PDPK1* (**c**). In turn, *PDPK1* negatively correlates with miR-423 (**d**). p-values below 0.05 are considered statistically significative.
