# Supplementary material for: Landscape of BRAF transcript variants in human cancer
Source: Mol Oncol. 2025 May 25;19(9):2700–14. doi: 10.1002/1878-0261.70043 (PMC12420348; doi:10.1002/1878-0261.70043)
Supplement: Supplementary file 2 — Table S1. Univariate Cox proportional hazards regression on 11 TCGA cancer types. [file MOL2-19-2700-s006.pdf]

Supplementary Table 1. Univariate Cox proportional hazards regression on 11 TCGA cancer types.

TCGA cancer types with BRAF mutation frequency higher than 4% (PMID: 35910024)			Univariate Cox proportional hazards regression					
**Cancer type (abbr)**	**Cancer type (extended)**	**n (subset used for hazards regression analysis)**	**variable**	**coef**	**HR**	**se(coef)**	**p-value**	**padj (BH)**
COAD	colon adenocarcinoma	260	Group2(vsGroup1)	0.00215	1.00215	0.28969	0.9941	0.999
			Group3(vsGroup1)	-0.56388	0.56900	0.32806	0.0856	0.3190
LUAD	lung adenocarcinoma	500	Group2(vsGroup1)	-0.06831	0.93397	0.18944	0.718	0.957
			Group3(vsGroup1)	0.23412	1.26379	0.17943	0.192	0.5280
SKCM	skin cutaneous melanoma	103	Group2(vsGroup1)	0.1295	1.1382	0.4697	0.783	0.957
			Group3(vsGroup1)	0.2222	1.2489	0.4784	0.642	0.9152
THCA	thyroid carcinoma	499	Group2(vsGroup1)	0.5784	17,832	0.5894	0.326	0.957
			Group3(vsGroup1)	-0.1630	0.8496	0.6712	0.808	0.9152
UCEC	uterine corpus endometrial carcinoma	175	Group2(vsGroup1)	-0.9498	0.3868	0.4847	0.06	0.660
			Group3(vsGroup1)	-0.2097	0.8109	0.3997	0.60	0.9152
**TCGA cancer types with BRAF CNV frequency higher than 60% (PMID: 35910024)**			**Univariate Cox proportional hazards regression**					
**Cancer type (abbr)**	**Cancer type (extended)**	**n (subset used for hazards regression analysis)**	**variable**	**coef**	**HR**	**se(coef)**	**p-value**	**padj (BH)**
ACC	adrenocortical carcinoma	79	Group2(vsGroup1)	-0.2671	0.7656	0.4287	0.533	0.957
			Group3(vsGroup1)	-0.8573	0.4243	0.5009	0.087	0.3190
ESCA	esophageal carcinoma	152	Group2(vsGroup1)	0.09724	1.10213	0.33469	0.771	0.957
			Group3(vsGroup1)	-0.09404	0.91025	0.32657	0.773	0.9152
GBM	glioblastoma multiforme	143	Group2(vsGroup1)	-0.15677	0.85490	0.23833	0.511	0.957
			Group3(vsGroup1)	0.04985	1.105111	0.23512	0.832	0.9152
**KIRP**	**kidney renal papillary cell carcinoma**	**250**	Group2(vsGroup1)	0.5379	1.7125	0.4655	0.24784	0.957
			**Group3(vsGroup1)**	**1.2287**	**3.4169**	**0.4279**	**0.004****	**0.04***
OV	ovarian serous cystadenocarcinoma	354	Group2(vsGroup1)	-0.10132	0.90365	-0.612	0.541	0.957
			Group3(vsGroup1)	-0.04274	0.95816	0.16422	0.795	0.9152
TGCT	testicular germ cell tumor	133	Group2(vsGroup1)	-2.11E+04	6.73E-07	2.29E+07	0.999	0.999
			Group3(vsGroup1)	-4.10E+04	1.56E-15	3.09E+07	0.999	0.9990

p-values: * <0.05, **<0.01
