# Supplementary material for: Landscape of BRAF transcript variants in human cancer
Source: Mol Oncol. 2025 May 25;19(9):2700–14. doi: 10.1002/1878-0261.70043 (PMC12420348; doi:10.1002/1878-0261.70043)
Supplement: Supplementary file 3 — Table S2. Brant Wald test on the proportional odds logistic regression for ordered category outcomes analysis performed on 250 KIRP patients. [file MOL2-19-2700-s003.pdf]

Supplementary Table 2. Brant Wald test on the proportional odds logistic regression for ordered category outcomes analysis performed on 250 KIRP patients.
Group1: patients with with the lowest *BRAF-204/BRAF-220* ratios (<=33rd percentile (1Q)).
Group2: patients with mid-range *BRAF-204/BRAF-220* ratios (>33rd percentile and <=66th percentile (2Q)).
Group3: patients with the highest *BRAF-204/BRAF-220* ratios (>66th percentile (3Q)).
DF: degrees of freedom, number of predictor variables minus one.

	Brant Wald Statistic	DF	p-value
Group2 vs Group1	3.7063374	4	0.4472049
Group3 vs Group1	3.6312914	2	0.1627328
Omnibus	0.3492023	2	0.8397919
