# Supplementary material for: Landscape of BRAF transcript variants in human cancer
Source: Mol Oncol. 2025 May 25;19(9):2700–14. doi: 10.1002/1878-0261.70043 (PMC12420348; doi:10.1002/1878-0261.70043)
Supplement: Supplementary file 4 — Table S3. TCGA cancer tissue samples (primary tumors). [file MOL2-19-2700-s007.pdf]

Supplementary Table 3. TCGA cancer tissue samples (primary tumors).

Cancer type		n
**abbr**	**extended**	
ACC	adrenocortical carcinoma	79
BLCA	bladder urothelial carcinoma	406
BRCA	breast invasive carcinoma	1095
CESC	cervical squamous cell carcinoma and endocervical adenocarcinoma	304
CHOL	cholangio carcinoma	35
COAD	colon adenocarcinoma	272
DLBC	lymphoid neoplasm diffuse large b-cell lymphoma	48
ESCA	esophageal carcinoma	162
GBM	glioblastoma multiforme	154
HNSC	head and neck squamous cell carcinoma	502
KICH	kidney chromophobe	65
KIRC	kidney renal clear cell carcinoma	532
KIRP	kidney renal papillary cell carcinoma	290
LAML	acute myeloid leukemia	150
LGG	brain lower grade glioma	514
LIHC	liver hepatocellular carcinoma	371
LUAD	lung adenocarcinoma	516
LUSC	lung squamous cell carcinoma	501
MESO	mesothelioma	87
OV	ovarian serous cystadenocarcinoma	376
PAAD	pancreatic adenocarcinoma	178
PCPG	pheochromocytoma and paraganglioma	165
PRAD	prostate adenocarcinoma	497
READ	rectum adenocarcinoma	94
SARC	sarcoma	259
SKCM	skin cutaneous melanoma	103
STAD	stomach adenocarcinoma	375
TGCT	testicular germ cell tumors	150
THCA	thyroid carcinoma	504
THYM	thymoma	120
UCEC	uterine corpus endometrial carcinoma	178
UCS	uterine carcinosarcoma	57
UVM	uveal melanoma	80
	**TOTAL**	**9219**
