# Supplementary material for: Landscape of BRAF transcript variants in human cancer
Source: Mol Oncol. 2025 May 25;19(9):2700–14. doi: 10.1002/1878-0261.70043 (PMC12420348; doi:10.1002/1878-0261.70043)
Supplement: Supplementary file 5 — Table S4. GTEx normal tissue samples. [file MOL2-19-2700-s008.pdf]

Supplementary Table 4. GTEx normal tissue samples.

Tissue type	n
Adrenal gland	100
Bladder	21
Brain	100
Breast	100
Cervix	19
Colon	100
Esophagus	100
Heart	100
Kidney	89
Liver	221
Lung	100
Muscle	100
Ovary	180
Pancreas	100
Prostate	241
Skin	100
Small intestine	187
Spleen	241
Stomach	100
Testis	100
Thyroid	100
Uterus	100
**TOTAL**	**2599**
