# Supplementary material for: Landscape of BRAF transcript variants in human cancer
Source: Mol Oncol. 2025 May 25;19(9):2700–14. doi: 10.1002/1878-0261.70043 (PMC12420348; doi:10.1002/1878-0261.70043)
Supplement: Supplementary file 6 — Table S5. Baseline characteristics of the 250 KIRP patients used for hazards regression analyses. [file MOL2-19-2700-s004.pdf]

Supplementary Table 5. Baseline characteristics of the 250 KIRP patients used for hazards regression analyses.

Variables	Total patients (n = 250)
**Survival Days**	
Mean ± SD	1021.076 ± 901.3952
Median (Range)	744 (0-5925)
**Clinical Stage**	
Stage I	166 (66.4%)
Stage II	21 (8.4%)
Stage III-Stage IV	63 (25.2%)
**Clinical T**	
T1-T2	195 (78%)
T3-T4	55 (22%)
**Clinical N**	
N0	223 (89.2%)
N1-N2	27 (10.8%)
**Clinical M**	
M0	241 (94.4%)
M1	9 (3.6%)
