# Supplementary material for: Landscape of BRAF transcript variants in human cancer
Source: Mol Oncol. 2025 May 25;19(9):2700–14. doi: 10.1002/1878-0261.70043 (PMC12420348; doi:10.1002/1878-0261.70043)
Supplement: Supplementary file 7 — Table S6. Correlation of BRAF‐204 with targeting miRNAs that are expressed in the KIRP dataset at TCGA. [file MOL2-19-2700-s002.pdf]

Supplementary Table 6. Correlation of *BRAF-204* with targeting miRNAs that are expressed in the KIRP dataset at TCGA.

miRNA ID	Spearman's rho correlation with *BRAF-204*	p-value	padj
**hsa-miR-3651**	**-0.2723647**	**2.51E-06**	**0.0002869**
**hsa-miR-423**	**-0.172104798**	**0.0033175**	**0.0498295**
hsa-miR-1260b	-0.109241021	0.0631922	0.3240235
hsa-miR-7704	-0.070576304	0.23084889	0.5769282
hsa-miR-320a	-0.041885364	0.47715958	0.7797102
hsa-miR-1260a	-0.009130528	0.87696403	0.9567465
hsa-miR-4792	0.004869664	0.93419367	0.9741557
hsa-miR-1246	0.064571864	0.27307254	0.6171108
