# Supplementary material for: Landscape of BRAF transcript variants in human cancer
Source: Mol Oncol. 2025 May 25;19(9):2700–14. doi: 10.1002/1878-0261.70043 (PMC12420348; doi:10.1002/1878-0261.70043)
Supplement: Supplementary file 8 — Table S7. Correlation of BRAF‐204 and miR‐423 with the experimentally validated targets of the miRNA that are expressed in the KIRP dataset at TCGA. [file MOL2-19-2700-s001.pdf]

Supplementary Table 7. Correlation of *BRAF-204* and miR-423 with the experimentally validated targets of the miRNA that are expressed in the KIRP dataset at TCGA.

gene name	gene symbol (HGNC)	Spearman's rho correlation with *BRAF-204*	p-value *BRAF-204*	padj *BRAF-204*	Spearman's rho correlation with miR-423	p-value miR-423	padj miR-423
**ENSG00000140992.18**	**PDPK1**	**0.727465895**	**2.22045E-16**	**1.07106E-15**	**-0.1894389**	**0.001234**	**0.003290667**
ENSG00000182621.17	PLCB1	0.376787774	4.88403E-11	1.8597E-10	-0.101397	0.0853	0.113733333
ENSG00000105698.15	USF2	0.29306119	4.4489E-07	1.27734E-06	-0.32351	2.20E-08	0.000000176
ENSG00000177732.8	SOX12	0.230804299	7.82011E-05	0.000182733	0.1683426	0.004147	0.0066352
ENSG00000138031.14	ADCY3	0.216130732	0.000220839	0.000491955	-0.2448067	2.27E-05	0.0000908
ENSG00000168884.14	TNIP2	0.131578372	0.025357285	0.04131975	-0.05742702	0.3304	0.3776
ENSG00000107562.16	CXCL12	0.131524679	0.025417523	0.041401748	0.1760366	0.002706	0.005412
ENSG00000071889.16	FAM3A	0.127984429	0.029671129	0.047605881	-0.02562691	0.6642	0.6642
